# Ideological polarization on anthropogenic climate change is stronger among politicians than among citizens across eight countries

**DOI:** 10.1038/s44458-026-00113-y

**Published:** 2026-07-14

**Authors:** Johannes Kotz, Helge Giese, Christian Breunig, Maj-Britt Sterba, Nathalie Brack, Patrick Dumont, Marija Taflaga, Javier Olivera, Lior Sheffer, Annika Werner, Anam Kuraishi, Wolfgang Gaissmaier

**Affiliations:** 1 Department of Psychology, University of Konstanz, Konstanz, Germany; 2 Cluster of Excellence “The Politics of Inequality”, University of Konstanz, Konstanz, Germany; 3 Charité - Universitätsmedizin Berlin, Berlin, Germany; 4 Department of Politics and Public Administration, University of Konstanz, Konstanz, Germany; 5 Université libre de Bruxelles, Brussels, Belgium; 6 Australian National University, Canberra, Australia; 7 Luxembourg Institute of Socio-Economic Research (LISER), Belval Esch-sur-Alzette, Luxembourg; 8 Pontificia Universidad Catolica del Peru, Lima, Peru; 9 Tel Aviv University, Tel Aviv, Israel; 10 University of Southampton, Southampton, UK; 11 University of Sussex, Brighton, UK

**Keywords:** Psychology and behaviour, Climate-change policy

## Abstract

Despite scientific consensus on anthropogenic climate change, political orientation is often associated with climate beliefs, and such polarization may hinder mitigation efforts. Yet, few studies directly compare politicians’ climate beliefs with those of citizens. Here, we used a large cross-national sample of politicians (*N* = 714) and citizens (*N* = 18,281) to explore how political orientation predicts climate beliefs and policy support in Australia, Belgium (Flanders and Wallonia), the Czech Republic, Germany, Israel, Luxembourg, the Netherlands, and Norway. Our results show that right-leaning politicians and citizens express weaker beliefs in anthropogenic climate change than left-leaning individuals. Ideological polarization is substantially stronger among politicians. Notably, right-leaning politicians are even less convinced about climate change than their own voters. These ideological divides are reflected in policy preferences: in both groups, belief in anthropogenic climate change statistically mediates the association between political orientation and support for mitigation policies, with markedly stronger mediation among politicians. Together, the results suggest that ideological gaps in climate beliefs, especially among politicians, may contribute to polarization in support for mitigation measures.

## Abstract (web summary)

Right-leaning politicians are even less convinced of anthropogenic climate change than their own voters, and ideological divides reflect policy support for climate mitigation policies, according to cross-national surveys in eight countries.

## Introduction

The scientific consensus on humans’ role in climate change is well-established, yet policies to mitigate anthropogenic climate change have not yet fully reflected this agreement^1^. Climate change skepticism remains a key barrier to mitigation, with many in developed democracies doubting human activities as the primary cause for climate change^2–4^. Tackling such skepticism could be especially effective among politicians since they are in a position to shape mitigation policies and influence public opinion. However, data on politicians’ beliefs in human-driven climate change compared to those of citizens and their relevance for mitigation actions are limited^5^. To assess potential mechanisms for strengthening mitigation efforts, it is essential to identify key factors associated with the belief in climate change and support for mitigation policies, particularly among policymakers.

Meta-analyses^6^ show that citizens’ beliefs in anthropogenic climate change are strongly linked to support for mitigation, although this effect weakens as policies become more specific. Indeed, climate change is a highly politicized topic in developed countries: ideologies and political orientation have a major impact on belief in human-driven climate change compared to smaller effects of other expected factors such as education and subjective knowledge. Belief in anthropogenic climate change has become politically polarized; that is, opposing groups have adopted increasingly divergent stances on the issue^7^. This polarization is evident among right-leaning individuals, who show substantially lower belief in climate change^6,8,9^ and lower trust in environmental scientists^10^ than left-leaning individuals.

While several studies have explored the effects of political orientation on belief in anthropogenic climate change in the public, their extent among politicians is less well-established. This is a major shortcoming because politicians have substantial direct influence over climate-related policy decision-making. Furthermore, politicians have indirect influence over public opinion polarization regarding climate change^11–14^. Therefore, it is important to assess how ideology affects politicians’ beliefs in climate change and whether these beliefs impact the support of concrete mitigation policies. As politicians have the responsibility to represent citizens’ preferences, it is also vital to compare the extent of polarization of belief in anthropogenic climate change and policy support among politicians and citizens, especially as stronger partisan polarization among politicians may also strengthen the public’s ideological divide^15^. Previous studies have found some ideological influences over the belief in climate change among Australian politicians^16^ and US policy advisors^17^; evidence from Belgium shows a mismatch between politicians and their electorate in support for mitigation policies, with less support among right-wing politicians than right-wing voters^18^. Despite these studies, researchers have, to our knowledge, yet to conduct cross-national comparisons of the political divide in belief in human-driven climate change between politicians and citizens, and its implications for policy support.

Using a large sample of politicians and citizens across eight developed democracies, we investigate in this study the role of political orientation in belief in human-driven climate change and its relationship with support for concrete climate mitigation policies. Since previous literature reported higher political polarization of belief in climate change in people with higher education and science literacy^19–24^, we control for the effects of education and the interaction between political orientation and education. Beyond the general relationship of political orientation with climate change beliefs, we investigate whether this association differs between politicians and citizens across countries. Finally, while controlling for education, we use mediation analyses to test whether belief in human-driven climate change mediates the relationship between political orientation and support for mitigation policies among politicians and citizens.

Our results show a clear difference between politicians and citizens in the political polarization of factual climate beliefs: politicians’ belief in human-driven climate change is even more strongly predicted by political orientation than that of citizens. This stronger polarization has direct implications for policy-making, as politicians’ climate beliefs mediate the relationship of their political orientation with support for mitigation policies.

## Results

### Sample description for citizens and politicians

Although the primary analyses of this study are conducted with a sample containing both citizens and politicians to enable direct comparisons between groups, we describe the citizen and politician samples separately in this section.

The citizen sample comprises 18,281 independent observations and was recruited to be representative for the respective country’s general population regarding gender, age and education, drawn from the official national statistics for each country. Participants were, on average, 48.6 (*SD* = 17.01) years old. 8,844 participants identified as male (48.4%), 9313 as female (50.9%), 67 as other gender (0.4%), and 57 participants did not provide an answer (0.3%). Among citizens, 14,048 participants (76.8%) had no university education, while 4,233 (23.2%) had a university education. The mean political orientation was 5.25 (*SD* = 2.37) on a Likert scale from 0 (Left-leaning) to 10 (Right-leaning), and, on average, citizens believed in anthropogenic climate change, with a mean of 5.13 (*SD* = 1.59) on a Likert scale from 1 (Strongly disagree) to 7 (Strongly agree). Descriptive statistics are listed in Table 1 for citizens and politicians and in Supplementary Table S1 for each country. Associations between demographics and other study variables were small or negligible (see Supplementary Table S2).

**Table 1:** Descriptive statistics of the study variables for the citizen and politician samples

	Citizens		Politicians	
Variable	*M*	SD	*M*	SD
1. Proportion non-male	0.51	0.50	0.40	0.49
2. Age/Proportion aged above median^a^	48.55	17.01	0.43	0.50
3. Proportion higher educated^b^	0.23	0.42	0.77	0.42
4. Political orientation^c^	5.25	2.37	4.66	2.53
5. Belief in climate change^d^	5.13	1.59	5.63	1.53
6. Support for increasing airplane ticket prices^e^	3.93	1.90	4.28	1.88
7. Support for subsidizing electric vehicles^f^	4.37	1.86	3.77	1.70

Means (*M*) and standard deviations (*SD*) of all study variables for the citizen and politician samples. All descriptive statistics were computed with the respective complete dataset for each variable. *N* for citizens varied between 17,635 and 18,281 participants and *N* for politicians varied between 530 and 714 participants.

^*a*^Citizens are reported with age in years and politicians with age categories coded as binary with 0 = Below median of the respective parliament and 1 = Above median of the respective parliament.

^*b*^Education level is coded as binary with 0 = Lower educated (no, primary, secondary and non-university tertiary education) and 1 = Higher educated (university education).

^*c*^Likert scale from 0 (Left) to 10 (Right).

^*d*,*e*,*f*^Likert scale from 1 (Strongly disagree) to 7 (Strongly agree).

The politician sample comprises 714 independent observations. All country teams of this project (except for Germany) made efforts to reach out to all national members of parliament via e-mail. In Germany, given the large parliament size, a sampled population of members of parliament at the national level was targeted, ensuring representativeness of parliament in terms of gender, party, and incumbent status. In addition, Belgium, a federalized country, included regional politicians in their target populations from Flanders and Wallonia. Supplementary Tables S3 and S4 display the representativeness of the full politician sample compared to their respective countries’ parliaments regarding gender, age, seniority, and party ideology. Gender and age distributions in our sample are similar to the target population with few exceptions (e.g., higher percentage of females in Israel). Seniority is overall slightly lower than in the respective parliaments, and we have a slightly lower participation rate among the right-wing in some countries. In the sample of this study, socio-demographic variables were pseudonymized to make individual politicians unidentifiable. Therefore, the age distribution is reported as 229 politicians above the median in the respective parliament (32.1%) and 301 below the median (42.2%). The age of 184 politicians was not publicly available, or they would have been identifiable based on this information and were thus assigned to a non-meaningful Other category (25.8%). The gender distribution was 380 male (53.2%), 253 female (35.4%), and 81 participants would have been identifiable based on their responses (11.3%). Among politicians, 164 (23%) had no university education, while 550 (77%) had a university education. The mean political orientation among politicians was 4.66 (*SD* = 2.53), and, with a mean of 5.63 (*SD* = 1.53), belief in anthropogenic climate change was higher than for citizens. Associations between demographics and the study variables were small or negligible (see Supplementary Table S5). Sample distributions for politicians and citizens across countries are presented in Table 2.

**Table 2:** Sample distribution across countries for politicians and citizens

	Full sample		Complete cases sample	
Country	Politicians	Citizens	Politicians	Citizens
Australia	57 (21%)^a^	1949	46	1804
Czechia	64 (32%)	2261	64	2074
Flanders (Belgium)	215 (85%)	2206	183	2065
Germany	177 (27%)	2242	158	2061
Israel	55 (32%)	2745	41	2581
Luxembourg	21 (36%)	1970	19	1910
Netherlands	38 (25%)	1942	33	1742
Norway	35 (21%)	2259	34	2061
Wallonia (Belgium)	148 (69.2%)	2238	136	1983
Total	810	22,123	714	18,281

Sample distribution across countries for politicians and citizens in the full sample and the sample used for main regression analyses.

^*a*^Response rate of politicians with the respective countries’ parliament sizes as target population.

### Stronger polarization of belief in anthropogenic climate change among politicians compared to citizens

We conducted unstandardized and standardized regression analyses on a sample that includes both politicians and citizens to examine the relationship of political orientation, education, and the interaction of the two with belief in anthropogenic climate change and compare those associations between politicians and citizens across countries (*N* = 18,995; see Supplementary Tables S6–S7 for detailed results). The regression models showed that political orientation was strongly associated with belief in anthropogenic climate change (*b* = − 0.24, *β* = − 0.35, *p* < 0.001), indicating that individuals with a stronger right-leaning political orientation had lower levels of climate change belief. Across the full political spectrum, this would predict a substantial difference of more than two points on a 7-point scale in belief in climate change between the most left (i.e., 0 on the political orientation scale) and the most right individuals (i.e., 10). This association was consistent across all countries; slopes for each country are shown in Supplementary Table S8. In contrast, education showed a negligible positive association with belief in climate change (*b* = 0.09, *β* = 0.05, *p* = 0.009). Differences in belief in climate change between politicians and citizens were also marginal, with slightly higher beliefs among politicians (*b* = 0.10, *β* = 0.06, *p* = 0.024).

**Fig. 1:**
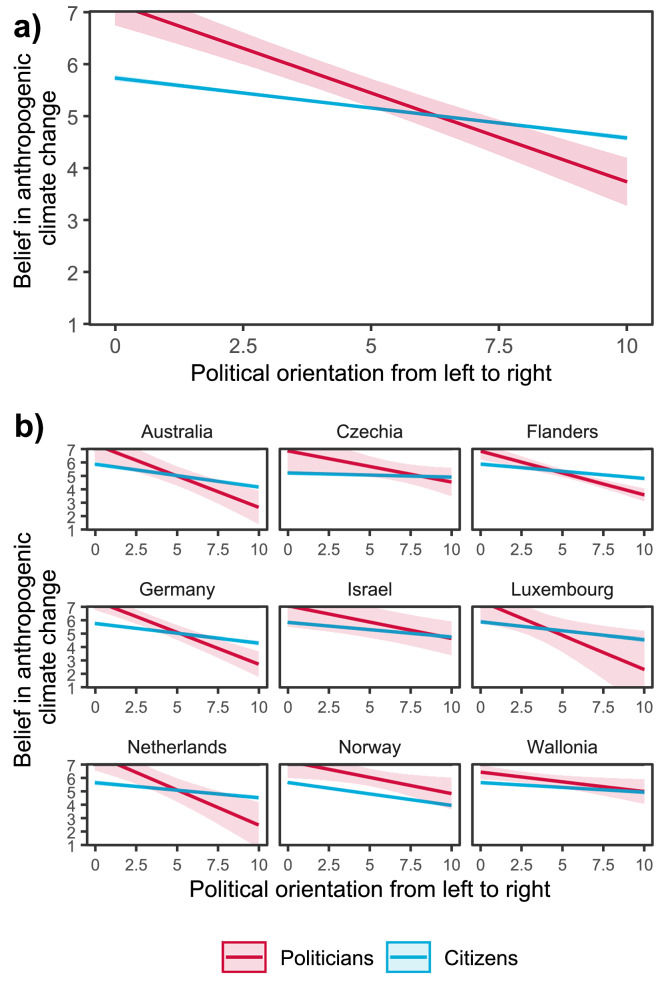
Predicted effects of political orientation on belief in anthropogenic climate change among politicians and citizens. **a** Predicted effects in the full sample. Fitted regression lines are shown with 95% confidence intervals (shaded areas) among politicians (*N * = 714; red line) and citizens (*N *= 18,281; blue line) based on the unstandardized regression model with robust standard errors used for analysis. **b** Predicted effects for each country.

However, the two-way interaction between political orientation and politician/citizen status indicates that political polarization of belief in anthropogenic climate change was stronger among politicians than citizens (*b* = − 0.12, *β* = − 0.18, *p* < 0.001), as illustrated in Fig. 1. Simple slope analyses confirmed a stronger association of political orientation with belief in climate change among politicians (*b* = − 0.35, *β* = − 0.53, *p* < 0.001) than among citizens (*b* = − 0.12, *β* = − 0.18, *p* < 0.001), and the slopes differed significantly between those groups (*t*(18,932) =  − 7.118, *p* < 0.001). Estimated marginal means and contrasts at  ± 1 standard deviation from the sample mean of political orientation (2.86 and 7.6 on a scale from 0 to 10; see Supplementary Table S9) further illustrate the divergence in the political orientation slope between politicians and citizens. While right-leaning citizens reported only slightly lower belief than left-leaning citizens (*Δ*_left−right_ = 0.56, *t*(18,932) = 17.307, *p* < 0.001), politicians exhibited a much steeper ideological divide, with substantially lower belief among right-leaning politicians and very strong belief among left-leaning politicians (*Δ*_left−right_ = 1.32, *t*(18,932) = 17.949, *p* < 0.001). This asymmetry was driven by divergence at both ends of the spectrum. On the left, politicians expressed stronger climate beliefs than citizens (*Δ*_politician−citizen_ = 0.752, *t*(18,932) = 10.157, *p* < 0.001), though both were on the higher end of the scale (*M*_Politicians,left_ = 6.21; *M*_Citizens,left_ = 5.46). On the right, the pattern reversed: Politicians expressed weaker belief in climate change than citizens (*Δ*_politician−citizen_ = − 0.362, *t*(18,932) = −2.460, *p* = 0.014), with both groups above but closer to the scale midpoint (*M*_Politicians,right_ = 4.53; *M*_Citizens,right_ = 4.89), suggesting lower acceptance of human-driven climate change. As Fig. 1 illustrates, the gaps between politicians and their electorate widened towards the ideological extremes.

The three-way interaction between political orientation, politician/citizen status and countries indicates that the difference in polarization between politicians and citizens varied across countries (Type-III F-Test for interaction between political orientation, group, and country: *F*_8,18932_ = 2.352, *p* = 0.016, see Supplementary Table S10). However, the direction of the interaction was consistent across countries in that slopes among politicians were steeper than those among citizens across all countries (see Supplementary Tables S11 and S12 for standardized slopes and contrasts between groups for each country). Based on model estimates with robust standard errors, Fig. 1 illustrates that polarization was stronger among politicians in all countries. However, in some countries, the group sizes of politicians were too small to make reliable statistical inferences.

Furthermore, we found no evidence for a two-way interaction between political orientation and education (*b* = − 0.00, *β* = − 0.01, *p* = 0.529), suggesting that the polarization of belief in climate change was not stronger among those with higher education levels. This supports literature questioning the role of cognitive sophistication and education in increasing political polarization of science beliefs^25^. Finally, analyses controlling for gender among both groups and age among citizens yielded similar results with negligible effects for these covariates (see Supplementary Tables S13–S16).

### Polarization of policy support is mediated by belief in anthropogenic climate change among politicians

We conducted path analyses for politicians and citizens separately to assess the role of belief in anthropogenic climate change in supporting concrete mitigation policies. In these analyses, belief in anthropogenic climate change served as a mediator between the independent variables political orientation and education, and the dependent variables support for increasing airplane ticket prices and subsidizing the purchase of electric vehicles.

**Fig. 2:**
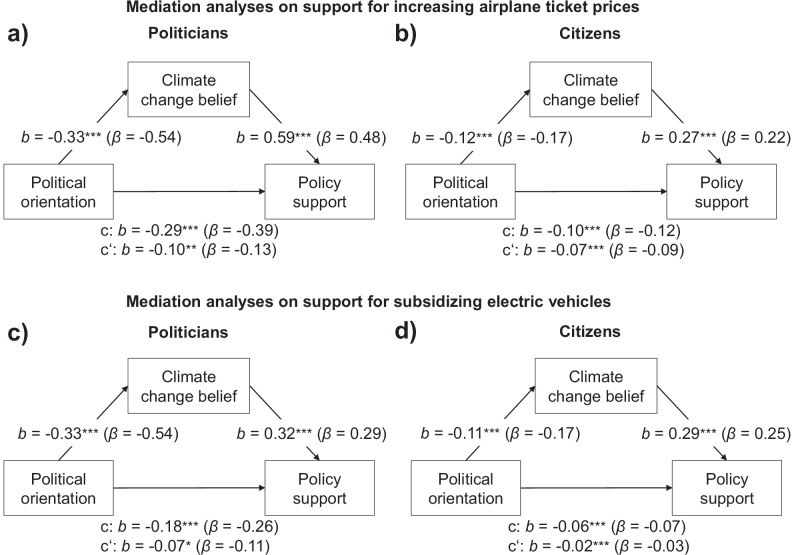
Path analyses with belief in anthropogenic climate change as mediator between political orientation and policy support in politicians and citizens. **a**, **b** Path analyses on support for increasing airplane ticket prices. **c**, **d** Path analyses on support for subsidizing electric vehicles. Panels (**a**, **c**) show the mediation paths for politicians (*N*_a)_ = 709; *N*_c)_ = 713) and panels (**b**, **d**) for citizens (*N*_b)_ = 17,696; *N*_d)_ = 17,635). Path coefficients are reported as unstandardized *b* and standardized *β*. Unstandardized and standardized total effects (c) and direct effects (c') of political orientation are reported under the direct path. Indirect effects are calculated as total effects (c) - direct effects (c'). Political orientation was grand-mean centered and *z*-standardized, with higher values indicating more right-leaning political orientation. ^*^ indicates *p* < 0.05. ^**^ indicates *p* < 0.01. ^***^indicates *p* < 0.001.

In the path analyses on policy support among politicians (*N* = 709 for increasing airplane ticket prices; *N* = 713 for subsidizing electric vehicles) shown in Fig. 2, belief in climate change was weaker among politicians with a right-leaning political orientation (coefficients for both policies were *b* = −0.33, *β* = − 0.54, all *p* < 0.001). Belief in anthropogenic climate change, however, had a strong and positive effect on support for mitigation policies (*b* = 0.59, *β* = 0.48, *p* < 0.001 for increasing airplane ticket prices and *b* = 0.32, *β* = 0.29, *p* < 0.001 for subsidizing electric vehicles). Political orientation only had a small direct effect on policy support, with right-leaning politicians showing less support for increasing airplane ticket prices (*b* = − 0.10, *β* = − 0.13, *p* = 0.001) and for subsidizing electric vehicles (*b* = − 0.07, *β* = − 0.11, *p* = 0.013). However, political orientation had an indirect effect on policy support through belief in climate change (*b* = − 0.19, *β* = − 0.26, *p* < 0.001 for higher airplane ticket prices and *b* = − 0.11, *β* = − 0.16, *p* < 0.001 for subsidizing electric vehicles), suggesting that politicians’ right-leaning orientation is indirectly associated with policy support via a weaker belief in climate change. Consequently, political orientation had a significant total effect on policy support among politicians (*b* = −0.29, *β* = −0.39, *p* < 0.001 for higher airplane ticket prices and *b* = −0.18, *β* = −0.26, *p* < 0.001 for subsidizing electric vehicles) with a partial mediation through belief in climate change. As already observed in our preceding analysis, belief in climate change was weaker among right-leaning politicians. This weaker belief, in turn, corresponded to lower support for both policies, given the positive association between climate change belief and policy support.

In both models, education showed minimal association with policy support, with no significant direct effects on policy support (*b* = 0.17, *β* = 0.09, *p* = 0.227 for increasing airplane ticket prices and *b* = − 0.13, *β* = − 0.07, *p* = 0.388 for subsidizing electric vehicles) and negligible indirect effects (*b* = 0.16, *β* = 0.08, *p* = 0.027 for increasing airplane ticket prices and *b* = 0.09, *β* = 0.05, *p* = 0.029 for subsidizing electric vehicles). Detailed results of the path analyses for politicians are shown in Supplementary Tables S17–S20. Additionally, path analyses including gender and age showed similar results (see Supplementary Tables S21 and S22).

In the path analyses on citizens’ policy support (*N* = 17,696 for increasing airplane ticket prices and *N* = 17,635 for subsidizing electric vehicles), the effect of political orientation on belief in climate change was smaller compared to politicians (*b* = − 0.12, *β* = −0.17, *p* < 0.001 for increasing airplane ticket prices and *b* = − 0.11, *β* = −0.17, *p* < 0.001 for subsidizing electric vehicles). However, similarly to politicians, belief in anthropogenic climate change showed a meaningful effect on policy support (*b* = 0.27, *β* = 0.22, *p* < 0.001 for increasing airplane ticket prices and *b* = 0.29, *β* = 0.25, *p* < 0.001 for subsidizing electric vehicles). The direct effect of political orientation on policy support was small for support of increasing airplane ticket prices (*b*= − 0.07, *β* = −0.09, *p* < 0.001) and very small for support of subsidizing electric vehicles (*b* = −0.02, *β* = −0.03, *p* < 0.001). The indirect effect of political orientation on both policy support items through belief in climate change was also very small in size although statistically significant (*b* = − 0.03, *β* = − 0.03, *p* < 0.001 for increasing airplane ticket prices and *b* = − 0.03, *β* = − 0.04, *p* < 0.001 for subsidizing electric vehicles). Among citizens, political orientation had only a small total effect on policy support (*b* = −0.10, *β* = −0.12, *p* < 0.001 for increasing airplane ticket prices and *b* = −0.06, *β* = −0.07, *p* < 0.001 for subsidizing electric vehicles). Although belief in climate change significantly mediated this relationship, the indirect effects were very small in magnitude, as shown in Fig. 2. In fact, mediation effects were substantially stronger among politicians. Contrasts between the indirect effects of political orientation among politicians and citizens, derived from multigroup structural equation models, showed stronger mediation effects for both policy support items in politicians (*b* = − 0.16, *β* = − 0.20, *p* < 0.001 for increasing airplane ticket prices and *b* = − 0.07, *β* = − 0.09, *p* < 0.001 for subsidizing electric vehicles).

Direct effects of education on both policy support items were not significant (*b* = − 0.06, *β* = −0.03, *p* = 0.075 for increasing airplane ticket prices and *b* = 0.02, *β* = 0.01, *p* = 0.444 for subsidizing electric vehicles) and indirect effects were negligible in size (*b* = 0.06, *β* = 0.03, *p* < 0.001 for increasing airplane ticket prices and *b* = 0.07, *β* = 0.04, *p* < 0.001 for subsidizing electric vehicles). Supplementary Tables S23–S30 show detailed results of the path analyses for citizens and multigroup SEMs. Analyses including gender and age showed similar results (see Supplementary Tables S31 and S32) with small direct effects of age on policy support (*b* = 0.01, *β* = 0.10, *p* < 0.001 for increasing airplane ticket prices and *b* = −0.01, *β* = −0.10, *p* < 0.001 for subsidizing electric vehicles), resulting in less support for increasing airplane ticket prices but more support for subsidizing electric vehicles among younger participants.

## Discussion

In this study, we used a large cross-national sample of politicians and citizens to examine the relationship between belief in anthropogenic climate change, political orientation, and education, as well as their associations with policy support. Consistent with previous literature^6^, political orientation was a much stronger predictor for belief in human-driven climate change than education among both politicians and citizens, suggesting that climate change is a politicized and polarizing issue. By utilizing cross-national comparisons between politicians and citizens, this study reveals that belief in anthropogenic climate change is more polarized among politicians than among citizens, with strong climate beliefs among left-leaning politicians but substantially lower belief in human-driven climate change among right-leaning politicians, who expressed even lower climate beliefs than right-leaning citizens. In mediation analyses on policy support, we showed that, although political orientation had a small direct effect on policy support among politicians, its major pathway was through belief in anthropogenic climate change. Among citizens, policy support was less tied to political orientation but directly associated with their belief in anthropogenic climate change. These findings highlight that belief in anthropogenic climate change plays a vital role in opinion formation, even for concrete political decisions made by both policymakers and citizens.

The substantially stronger political polarization of belief in anthropogenic climate change among politicians compared to citizens suggests a mismatch between politicians and their voters on climate change views. This stronger polarization among politicians was consistently found across countries, showing that it is not merely a local issue. Realigning right-leaning politicians’ positions with those of their electorate in particular could foster cross-party consensus on climate action and reduce polarization on climate change globally. Although politicians have more accurately informed beliefs about political issues than citizens^26^, they still exhibit strong partisan polarization of these beliefs^26^ and are biased by prior attitudes^27^. Furthermore, they seem to be more resistant to debiasing interventions than citizens^28^. However, elected representatives should be responsive to the deviating opinions of their electorate^29,30^. Since politicians are rather inaccurate in estimating policy preferences of their own electorate^31^ and underestimate public support for climate action^18,32,33^, it could be beneficial to inform politicians, especially on the right wing, about voters’ actual beliefs and attitudes to reduce such biases and encourage them to act accordingly.

However, beyond misperception of public opinion^34,35^ or stronger ideological influences on issue attitudes^36^, the stronger polarization of climate beliefs among politicians may also reflect strategic position-taking rather than actual epistemic beliefs about climate change. Politicians face incentive structures such as party constraints, electoral incentives, or lobbying pressures^37,38^ that citizens do not have when stating opinions, potentially leading to more extreme expressions of climate opinions regardless of their personal assessment of the science.

Such polarized elite positions can shape voters’ attitudes about climate change^11,39^ and drive ideological and affective polarization among the public as well, particularly among politically interested citizens^40,41^. Our findings that politicians are more polarized than citizens are consistent with models of top-down opinion formation^15^ predicting that elite cues influence citizen opinion, without citizens fully mirroring elite positions. Citizens form political attitudes through multiple channels beyond elite cues, including socialization, issue salience, personal experience, other sources of information and self-interest, which attenuates the degree to which elite-level polarization is transmitted to the public^42–44^.

Nevertheless, increasingly partisan elite positions appear to have contributed to public skepticism towards climate science^12^. Addressing the political incentives and ideological constraints that are potentially driving elite polarization requires structural solutions alongside epistemic ones, such as reducing financial and lobbying incentives for climate skepticism, or reforming electoral systems to reduce re-election pressures that reward partisan positioning. Informing right-leaning citizens that their representatives’ climate positions diverge from their own could further encourage them to pressure politicians or vote accordingly, increasing congruence between elite positions and voters’ preferences.

Reducing elite polarization of belief in anthropogenic climate change is especially important in light of its mediating role between political orientation and policy support found in this study. Politicians’ attitudes towards mitigation policies were politically polarized but heavily associated with their viewpoints on climate change. Improving the consensus on climate science across the political spectrum, combined with exposure to voters’ stances and structural reforms to reduce political incentives, could therefore be a key lever for enabling science-based political debates about climate action that focus on the issue rather than on the political divide of underlying beliefs. For citizens, mitigation action was a less politicized issue and was directly associated with belief in human-driven climate change. While previous findings have identified beliefs about the effectiveness of policies and their financial impact on the own and lower-income households to be strong predictors for policy support^45^, we find that strengthening belief in climate change is also important for fostering science-based opinions about climate action among the public.

The strong political polarization of belief in human-driven climate change and its relevance for policy support hold across various demographics, as education, gender, and age only had minimal effects. Furthermore, we found no evidence that the association of political orientation with belief in anthropogenic climate change is stronger among the higher educated as proposed by literature on identity-protective cognition^20,21^. This is, however, consistent with recently found negligible effects of cognitive sophistication on polarization of science beliefs^25^. Consequently, the risk of increasing polarization of beliefs through educating citizens about climate change seems low. Instead, recent literature has identified promising informational interventions for depolarizing climate change perceptions among the public, such as emphasizing effective collective action^9^, reducing psychological distance of climate change impacts^9,46^, educating about climate science^47,48^, or communicating the scientific consensus on climate change^49,50^. Beyond merely informing citizens, it is also important to overcome ideological barriers such as motivated partisan cognition and group identity norms^51^. Reducing the impact of partisan identities through framing climate action as value-congruent seems to be a promising approach to reduce polarization of climate opinions^52–54^. However, given the even stronger polarization we found in this study among politicians and their political incentives possibly influencing climate opinions, it needs to be tested whether such interventions can also improve climate beliefs and policy support among political elites across the ideological spectrum.

This study’s results are based on a large cross-national sample of politicians and citizens, but some limitations should be noted. First, the sample size of politicians is smaller than that of citizens, leading to wider estimates among politicians. However, using heteroskedasticity-consistent covariance matrices for regression analyses adjusts for these unequal group sizes in our analyses. Second, the binary measure for education might underestimate education and interaction effects, and the overall high education level among politicians complicates comparisons by education level. Nonetheless, patterns of polarization and education effects across countries are consistent with existing literature on country-comparisons of climate change perceptions^55^. Third, due to the pseudonymization of politicians, demographic data in the analyses were limited, and potential confounding by other socio-demographic factors cannot be ruled out. However, their impact is known to be much smaller than that of ideological factors^6^. Finally, this study uses observational data. Therefore, causal pathways in mediation analyses are theoretical assumptions, justified by a broader influence of political orientation on support for mitigation policies compared to a more direct impact of the closely related belief in climate change. This causal ordering of both climate change perceptions and political orientation shaping support for climate action is an established theoretical approach in the literature on citizens^6,56–58^. However, among politicians, if expressed climate beliefs reflect strategic positioning in line with party stances on climate policy rather than genuine epistemic states, then policy preferences may drive expressed climate beliefs rather than the reverse. Since our observational data cannot distinguish between these two causal orderings, we applied the same theoretical framework to both politicians and citizens to keep the mediation analyses comparable. Future research using experimental designs could help establish the direction of this relationship among political elites.

In conclusion, we show that belief in anthropogenic climate change is politically polarized and relevant for the support of mitigation policies among politicians and citizens across developed democracies. With even stronger polarization of belief in human-driven climate change and its relevance for policy support among politicians, this study highlights the need to develop effective communication strategies and structural reforms to foster science-based and nonpartisan debates in politics and realign politicians’ and voters’ views about climate change, especially among right-wing politicians.

## Methods

### Sample

Our data included national and regional politicians and citizens from eight developed democracies: Australia, Belgium (Flanders and Wallonia reported separately as they have different political systems), Czechia, Germany, Israel, Luxembourg, the Netherlands, and Norway. In total, *N* = 22,933 participants completed the survey containing questions about their belief in anthropogenic climate change and support for mitigation policies. This includes *n* = 810 national and regional politicians and *n* = 22,123 citizens. The citizen surveys are representative of their respective countries regarding gender, age, and education. Since we conducted the analyses only with complete cases for belief in human-driven climate change, political orientation, and education, the final sample used in the linear regression model to identify differences between groups consists of *n* = 714 politicians and *n* = 18,281 citizens. We conducted subsequent path analyses to identify the relationships with policy support with complete cases only; we report the final sample sizes with the analyses’ results.

### Study procedures

The data collection for this study was part of the second wave of the ERC-funded project ’How Politicians Evaluate Public Opinion’ (POLPOP II), hosted by the University of Antwerp, which gathered data from citizens and politicians across thirteen developed democracies through an online survey. Since belief in anthropogenic climate change was not assessed in all countries, we report data from the eight countries in this study. Data collection for politicians was conducted between February 2022 and May 2023 by research teams in the respective countries. Participating politicians were asked to answer a thirty-minute questionnaire programmed in Qualtrics. In all countries, we made sure that surveys were taken by politicians in the presence of a researcher, either in person, online, or occasionally by phone. The data collection for citizens was coordinated by the University of Antwerp and conducted by the panel provider Dynata between February 2022 and May 2022. Approval for conducting the surveys with politicians was obtained from the ethics committees of the respective universities in all countries (see Supplementary Table S33 for details about ethics clearance in each country). Approval for the citizen surveys was obtained from the Ethics Committee for the Social Sciences and Humanities at the University of Antwerp. Participants provided informed consent before completing the survey, which contained socio-demographic variables and several blocks about political attitudes, behavior, and their opinions on various topics. From this survey, we report results on age, gender, education, political orientation, belief in anthropogenic climate change, and support for policies to mitigate climate change.

### Measures

As some politicians could have been identified based on party affiliation and socio-demographic data, age and gender variables were pseudonymized to protect politicians’ anonymity. Age was divided into two categories based on the respective parliaments’ median age, and gender was divided into male and female participants. For both demographic variables, a third non-meaningful Other category was created to group data from politicians who could otherwise be identifiable. This category contains 184 politicians for the age category and 81 for gender and was not included in analyses controlling for demographics. In the citizen sample, we calculated respondents’ age based on their indicated birth year. Gender was assessed with Male, Female, and Other as answer options, and we included 67 participants reporting other genders in the Female category for analyses, following an approach supported in the literature^59^.

Education included four categories adapted to the education systems of the respective countries: No or Primary Education, Secondary Education, Higher Non-University Education, and University Education. For the purposes of this study and to make the results comparable between countries, we combined these categories into two dichotomous groups, with Lower education combining the first three categories and Higher education for the university education category.

Political orientation was measured with a self-report item on an 11-point Likert scale, ranging from 0 to 10, with Left and Right as respective anchors. Therefore, a higher score on the scale indicates a more right-leaning self-reported political orientation.

Belief in anthropogenic climate change was measured by asking participants to rate their agreement with the statement ’Climate change is mostly due to human activity’ on a 7-point Likert scale, ranging from Strongly Disagree to Strongly Agree.

Support for policies to mitigate climate change was measured using two items from the policy preference block of the multi-country POLPOP II survey. Participants rated their agreement with statements about increasing the prices of airplane tickets to reduce carbon emissions and subsidizing electric vehicle purchases on a 7-point Likert scale, ranging from Strongly Disagree to Strongly Agree. Full wordings of all items are reported in Supplementary Table S34.

### Analyses

For the statistical analyses, we used R-4.3.1^60^ with the packages emmeans^61^ for post-hoc analyses of linear regression models, lavaan^62^ for path models, and stats for linear regression analyses. To compare the relationships of political orientation and education with belief in anthropogenic climate change between politicians and citizens across countries, we conducted unstandardized and standardized linear regression models on belief in anthropogenic climate change with group (Politician/Citizen) and country as moderators. Furthermore, we introduced an interaction term between political orientation and education to analyze whether education level moderates the association of political orientation with belief in anthropogenic climate change. To assess the heterogeneity of effects between countries, we conducted Type-III F-Tests for interactions with country as moderator in the regressions. Furthermore, country was included as an effect-coded factor in all regression models, weighting each country equally regardless of sample size and ensuring that estimates are interpreted relative to the grand mean across countries. To adjust for the different group sizes between countries and between politicians and citizens, standard errors were corrected by calculating heteroscedasticity-consistent covariance matrices for all regressions. Based on the linear regression models, we conducted simple slope analyses comparing slopes of political orientation associations between politicians and citizens. Furthermore, we estimated marginal means at  ± 1 standard deviation around the sample mean of political orientation for politicians and citizens separately and conducted contrast analyses between left-leaning politicians and citizens and between right-leaning politicians and citizens. Finally, we analyzed the relationship of belief in anthropogenic climate change and its antecedents with the support for policies to mitigate climate change by using path analyses for both groups and for each policy support item separately. We treated belief in anthropogenic climate change as a mediator between policy support as the dependent and political orientation and education as the independent variables. To test for differences in mediation effects between politicians and citizens, we conducted multigroup structural equation models with contrasts between indirect effects for both groups.

We conducted all analyses with effect-coded factors, and we used *z*-standardized continuous variables for standardized regressions. For unstandardized regressions, we used the original measure scales, except for grand-mean centered political orientation, to make their results comparable to standardized regression results. For the path analyses, we used 10,000 bias-corrected bootstrap confidence intervals. All statistical tests were two-sided, and we used a *p*-value of 0.05 for statistical inferences. We report *β* as the standardized effect size measure throughout the results. Since age was assessed differently among politicians than among citizens, this variable was not introduced in the linear regression model comparing groups. Furthermore, we excluded gender and age categories from the path analyses containing politicians’ data because their inclusion would substantially reduce the sample size by 220 politicians due to pseudonymization. To make analyses comparable between groups, we also excluded gender and age from the path analyses with citizens. However, we additionally analyzed linear regression models that included gender for both groups and age for citizens, as well as path analyses that included gender and age categories for politicians and gender and age for citizens.

### Reporting summary

Further information on research design is available in the Nature Research Reporting Summary linked to this article.

## Supplementary information

Transparent Peer Review file

Supplementary Information

Reporting Summary

## Data Availability

The data and materials^63^ used in this study are openly available on Open Science Framework (10.17605/OSF.IO/H625R).

## Code availability

The analysis code^63^ used in this study is openly available on Open Science Framework (10.17605/OSF.IO/H625R).
