# Supplementary material for: Ideological polarization on anthropogenic climate change is stronger among politicians than among citizens across eight countries
Source: Commun Sustain. 2026 Jul 14;1(1):111. doi: 10.1038/s44458-026-00113-y (PMC13368580; doi:10.1038/s44458-026-00113-y)
Supplement: Supplementary file 2 — Supplementary Information [file 44458_2026_113_MOESM2_ESM.pdf]

Supplementary Information for

Ideological polarization on anthropogenic climate change is stronger among politicians than among citizens across eight countries

Johannes Kotz, Helge Giese, Christian Breunig, Maj-Britt Sterba, Nathalie Brack, Patrick Dumont, Marija Taflaga, Javier Olivera, Lior Sheffer, Annika Werner, Anam Kuraishi, Wolfgang Gaissmaier

Corresponding author: Johannes Kotz.
E-mail: johannes.kotz@uni-konstanz.de

This PDF file includes:

Tables S1 to S34

Supplementary Table S1

Table S1. Descriptive statistics of the study variables for politicians and citizens in each country

Countries	Prop. non-males		Age/Prop. over median[a]		Prop. higher edu.[b]		Pol. or.[c]		Climate belief		Support: Airpl. tick.[e]		Support: EV subsidy[f]	
	M	*SD*	*M*	*SD*	*M*	*SD*	*M*	*SD*	*M*	*SD*	*M*	*SD*	*M*	*SD*
Australia														
Politicians	0.41	0.5	0.57	0.5	0.78	0.42	4.48	2.28	5.46	1.72	3.37	1.69	3.93	1.94
Citizens	0.55	0.5	47.89	17.41	0.21	0.41	5.3	2.26	4.96	1.72	3.73	1.82	4.83	1.78
Czechia														
Politicians	0.31	0.46	0.22	0.42	0.83	0.38	6.86	1.69	5.12	1.12	3.44	1.49	3.09	1.54
Citizens	0.53	0.5	47.84	16.39	0.18	0.39	5.41	2.16	5.06	1.53	3.81	1.71	3.95	1.88
Flanders														
Politicians	0.42	0.5	0.51	0.5	0.66	0.48	5.37	2.51	5.27	1.67	4.19	1.94	3.44	1.61
Citizens	0.51	0.5	48.9	16.73	0.2	0.4	5.44	2.3	5.3	1.48	4.47	1.83	4.58	1.83
Germany														
Politicians	0.4	0.49	0.38	0.49	0.88	0.33	4.23	2.11	5.77	1.64	4.5	1.83	3.89	1.75
Citizens	0.52	0.5	51.26	17.03	0.23	0.42	4.62	1.91	5.1	1.63	4.54	1.93	4.24	1.85
Israel														
Politicians	0.27	0.45	0.48	0.51	0.9	0.3	4.78	3.08	5.51	1.25	2.8	1.52	4.62	1.76
Citizens	0.53	0.5	42.96	15.92	0.37	0.48	6.08	2.71	5.19	1.54	2.85	1.64	4.59	1.76
Luxembourg														
Politicians	0.33	0.49	0.46	0.52	0.74	0.45	3.68	1.86	5.74	1.52	4.63	2.03	4.95	1.75
Citizens	0.48	0.5	48.71	16.4	0.34	0.47	4.72	1.89	5.3	1.54	4.01	1.82	4.09	1.85
Netherlands														
Politicians	0.55	0.51	0.52	0.51	0.76	0.44	3.82	2.35	5.64	1.87	5.15	2.14	4.21	1.78
Citizens	0.55	0.5	53.54	17	0.08	0.27	5.28	2.32	5.07	1.54	4.63	1.83	4.78	1.77
Norway														
Politicians	0.41	0.5	0.54	0.51	0.62	0.49	5.15	2.55	6.09	1.33	4.79	1.57	4.76	1.21
Citizens	0.47	0.5	49.21	17.55	0.28	0.45	5.22	2.66	4.82	1.73	3.46	1.84	3.84	1.9
Wallonia														
Politicians	0.42	0.5	0.43	0.5	0.77	0.42	3.43	2.38	6.18	1.06	4.89	1.7	3.58	1.53
Citizens	0.49	0.5	48.78	16.82	0.13	0.34	4.94	2.43	5.32	1.53	4.23	1.93	4.52	1.87

Note. Means (*M*), standard deviations (*SD*) of all study variables for the politician ($N = 714$) and citizen ($N = 18{,}281$) samples for each country. Country-specific sample sizes are listed in Table S1.
[a]Binary coded for politicians with 0 = Below median of the respective parliament and 1 = Above median of the respective parliament. [b]Binary coded with 0 = Lower educated and 1 = Higher educated. [c]Likert scale from 0 (Left) to 10 (Right). [d,e,f]Likert scale from 1 (Strongly disagree) to 7 (Strongly agree).

Johannes Kotz, Helge Giese, Christian Breunig, Maj-Britt Sterba, Nathalie Brack, Patrick Dumont, Marija Taflaga, Javier Olivera, Lior Sheffer, Annika Werner, Anam Kuraishi, Wolfgang Gaissmaier

Supplementary Table S2

Table S2. Descriptive statistics of the study variables and correlations between them for the citizen sample

Variable	*M*	*SD*	1	2	3	4	5	6
1. Proportion of non-male participants	0.51	0.50						
2. Age	48.55	17.01	-.07**					
3. Proportion of higher educated[a]	0.23	0.42	-.01	-.13**				
4. Political orientation[b]	5.25	2.37	-.07**	-.02**	-.01			
5. Belief in climate change[c]	5.13	1.59	.05**	-.05**	.06**	-.17**		
6. Support for increasing airplane ticket prices[d]	3.93	1.90	-.02**	.10**	.00	-.12**	.24**	
7. Support for subsidizing electric vehicles[e]	4.37	1.86	.00	-.11**	.02**	-.07**	.26**	.15**

Note. Means (*M*), standard deviations (*SD*) and pearson's correlations of all study variables for the citizen sample. All correlations were computed with the respective complete dataset and *N* varies between 17,233 and 18,281 participants.
[a]Binary coded with 0 = Lower educated and 1 = Higher educated. [b]Likert scale from 0 (Left) to 10 (Right). [c,d,e]Likert scale from 1 (Strongly disagree) to 7 (Strongly agree).
* indicates $p < .05$. ** indicates $p < .01$.

Supplementary Table S3

Table S3. Representativity of MPs who cooperated compared with the MP population for gender, age and seniority

	Australia		Czechia		Flanders (BE)	
Variable	Participants	Population	Participants	Population	Participants	Population
Total N	58 (21%)	273	64 (32%)	200	215 (85%)	254
Female	20 (34.5%)	109 (39.9%)	21 (33%)	52 (26%)	89 (41%)	115 (45%)
Age in years (SD)	53.6 (9.6)	52.3 (9.6)	48.45 (9.6)	52.13 (9.5)	47.5 (9.2)	47.4 (8.95)
Seniority in years (SD)	7.7 (7.4)	8.7 (7.7)	4.8 (5.15)	5.7 (4.7)	9.0 (7.4)	9.1 (7.4)

	Germany		Israel		Luxembourg	
Variable	Participants	Population	Participants	Population	Participants	Population
Total N	178 (27%)	738[a]	55 (32%)	174	21 (36%)	60
Female	72 (41%)	258 (35%)	17 (74%)	36 (21%)	7 (33%)	21 (35%)
Age in years (SD)	46.8 (12.0)	48.5 (11.1)	55 (10.2)	54.4 (10.9)	52.7	53.8
Seniority in years (SD)	6.2 (6.5)	8.6 (7.8)	6.3 (7.2)	8.2 (7.9)	9.95	12.15

	Netherlands		Norway		Wallonia (BE)	
Variable	Participants	Population	Participants	Population	Participants	Population
Total N	38 (25%)	152	35 (21%)	169	148 (69.2%)	214
Female	21 (55%)	59 (39%)	11 (31%)	76 (45%)	60 (40.5%)	86 (40.2%)
Age in years (SD)	45.2 (7.5)	46.4 (9.2)	47.6 (12.7)	47.5 (11.4)	47.4 (9.8)	49.0 (10.5)
Seniority in years (SD)	4.2 (3.5)	6.4 (5.7)	4.9 (4.6)	7.5 (7.1)	6.4 (6.1)	7.5 (7.0)

Note. $N = 812$. Comparison of gender, age and seniority distributions between politicians who participated in the survey and the population of politicians in the respective countries' parliament. The table shows aggregated data before pseudonymization and stems from the Fieldwork Report of the POLPOP II project.
[a] Actual number of contacted politicians in Germany = 658 (sampling).

 Johannes Kotz, Helge Giese, Christian Breunig, Maj-Britt Sterba, Nathalie Brack, Patrick Dumont, Marija Taflaga, Javier Olivera, Lior Sheffer, Annika Werner, Anam Kuraishi, Wolfgang Gaissmaier

Supplementary Table S4

Table S4. Representativity of MPs who cooperated compared with the population for party ideology

	Australia[a]		Czechia		Flanders (BE)	
Variable	Participants	Population	Participants	Population	Participants	Population
Total *N*	58 (21%)	273	64 (32%)	200	215 (85%)	254
Left (CHES 0-3)	24 (41.4%)	129 (47.2%)	0 (0%)	0 (0%)	61 (28%)	67 (26%)
Centre (CHES 4-6)	4 (6.9%)	16 (5.9%)	45 (70%)	132 (66%)	62 (29%)	75 (30%)
Right (CHES 7-10)	30 (51.7%)	127 (46.5%)	19 (30%)	68 (34%)	89 (41%)	108 (43%)
Other	/	/	/	/	3 (1%)	4 (2%)

	Germany		Israel		Luxembourg	
Variable	Participants	Population	Participants	Population	Participants	Population
Total *N*	178 (27%)	738[b]	55 (32%)	174	21 (36%)	60
Left (CHES 0-3)	103 (57.9%)	364 (49.3%)	17 (31%)	63 (38%)	2 (10%)	2 (3%)
Centre (CHES 4-6)	46 (25.8%)	245 (33.2%)	7 (13%)	23 (14%)	12 (57%)	33 (55%)
Right (CHES 7-10)	28 (15.7%)	125 (16.9%)	31 (57%)	80 (48%)	7 (33%)	25 (42%)
Other	1 (0.01%)	4 (0.01%)	/	/	/	/

	Netherlands		Norway		Wallonia (BE)	
Variable	Participants	Population	Participants	Population	Participants	Population
Total *N*	38 (25%)	152	35 (21%)	169	148 (69.2%)	214
Left (CHES 0-3)	11 (28.95%)	34 (22.4%)	15 (43%)	72 (43%)	94 (63.5%)	126 (58.9%)
Centre (CHES 4-6)	16 (42.11%)	51 (33.6%)	19 (54%)	75 (44%)	22 (14.9%)	33 (15.4%)
Right (CHES 7-10)	11 (28.95%)	67 (44%)	1 (3%)	21 (12%)	31 (20.9%)	52 (24.3%)
Other	/	/	0 (0%)	1 (1%)	1 (0.7%)	3 (1.4%)

Note. $N = 812$. Comparison of party ideology between politicians who participated in the survey and the population of politicians in the respective countries' parliament. This comparison relies on the most recent Chapel Hill Expert Survey (CHES) for data about parties' positions on ideology and policy issues ranging from 0 to 10. The table shows aggregated data before pseudonymization and stems from the Fieldwork Report of the POLPOP II project.
[a]There is no CHES data available about Australian parties but local researchers classified parties on the same axis.
[b]Actual number of contacted politicians in Germany = 658 (sampling).

Supplementary Table S5

Table S5. Descriptive statistics of the study variables and correlations between them for the politician sample

Variable	*M*	*SD*	1	2	3	4	5	6
1. Proportion of non-male participants	0.40	0.49						
2. Proportion aged above median[a]	0.43	0.50	-.06					
3. Proportion of higher educated[b]	0.77	0.42	.03	-.19**				
4. Political orientation[c]	4.66	2.53	-.15**	.08	-.05			
5. Belief in climate change[d]	5.63	1.53	.15**	-.15**	.10**	-.54**		
6. Support for increasing airplane ticket prices[e]	4.28	1.88	.15**	-.06	.09*	-.39**	.55**	
7. Support for subsidizing electric vehicles[f]	3.77	1.70	.07	-.03	.00	-.26**	.34**	.26**

Note. Means (M), standard deviations (SD) and pearson's correlations of all study variables for the politician sample. All correlations were computed with the respective complete dataset and N varies between 494 and 714 participants.
[a]Binary coded with 0 = Below median of the respective parliament and 1 = Above median of the respective parliament. [b]Binary coded with 0 = Lower educated and 1 = Higher educated. [c]Likert scale from 0 (Left) to 10 (Right). [d,e,f]Likert scale from 1 (Strongly disagree) to 7 (Strongly agree).
* indicates $p < .05$. ** indicates $p < .01$.

Supplementary Table S6

Table S6. Results of unstandardized regression model on belief in climate change moderated by groups and countries

	Belief in climate change			
Predictors	*b*	*SE*[a]	*95% CI*	*p*
Main effects				
Intercept	5.27	0.04	[5.19; 5.36]	<.001
Politician[b]	0.10	0.04	[0.01; 0.18]	.024
Education level[c]	0.09	0.03	[0.02; 0.15]	.009
Political orientation[d]	−0.24	0.02	[-0.27; -0.20]	<.001
Education level x Politician	−0.02	0.03	[-0.09; 0.04]	.483
Political orientation x Politician	−0.12	0.02	[-0.15; -0.09]	<.001
Political orientation x Education level	0.00	0.01	[-0.02; 0.01]	.529
Covariates				
Country: Australia[e]	−0.20	0.13	[-0.46; 0.05]	.115
Country: Czechia	0.06	0.10	[-0.14; 0.25]	.569
Country: Flanders	0.03	0.07	[-0.10; 0.17]	.610
Country: Germany	−0.20	0.09	[-0.39; -0.02]	.027
Country: Israel	0.20	0.09	[0.02; 0.37]	.026
Country: Luxembourg	−0.19	0.20	[-0.59; 0.21]	.346
Country: Netherlands	−0.22	0.17	[-0.55; 0.11]	.195
Country: Norway	0.24	0.10	[0.04; 0.44]	.019
Politician x Country: Australia	−0.15	0.13	[-0.40; 0.10]	.250
Politician x Country: Czechia	0.19	0.10	[-0.00; 0.38]	.050
Politician x Country: Flanders	−0.17	0.07	[-0.30; -0.03]	.014
Politician x Country: Germany	−0.05	0.09	[-0.24; 0.13]	.565
Politician x Country: Israel	0.09	0.09	[-0.09; 0.26]	.332
Politician x Country: Luxembourg	−0.33	0.20	[-0.72; 0.07]	.108
Politician x Country: Netherlands	−0.17	0.17	[-0.50; 0.16]	.310
Politician x Country: Norway	0.44	0.10	[0.24; 0.64]	<.001
Education level x Country: Australia	0.16	0.12	[-0.08; 0.39]	.202
Education level x Country: Czechia	−0.16	0.09	[-0.32; 0.01]	.068
Education level x Country: Flanders	0.05	0.06	[-0.07; 0.18]	.396
Education level x Country: Germany	0.04	0.08	[-0.11; 0.18]	.627
Education level x Country: Israel	−0.24	0.09	[-0.42; -0.06]	.009
Education level x Country: Luxembourg	0.11	0.11	[-0.10; 0.33]	.291
Education level x Country: Netherlands	−0.10	0.12	[-0.33; 0.13]	.381
Education level x Country: Norway	0.12	0.10	[-0.07; 0.30]	.229
Political orientation x Country: Australia	−0.06	0.05	[-0.16; 0.03]	.172
Political orientation x Country: Czechia	0.09	0.04	[0.01; 0.17]	.031
Political orientation x Country: Flanders	0.00	0.03	[-0.05; 0.05]	.945
Political orientation x Country: Germany	−0.08	0.03	[-0.14; -0.02]	.012
Political orientation x Country: Israel	0.06	0.03	[0.00; 0.11]	.032
Political orientation x Country: Luxembourg	−0.09	0.08	[-0.25; 0.07]	.260
Political orientation x Country: Netherlands	−0.07	0.05	[-0.18; 0.04]	.195
Political orientation x Country: Norway	0.03	0.04	[-0.05; 0.12]	.464
Political orientation x Politician x Country: Australia	−0.03	0.05	[-0.12; 0.06]	.503
Political orientation x Politician x Country: Czechia	0.02	0.04	[-0.07; 0.10]	.713
Political orientation x Politician x Country: Flanders	0.01	0.03	[-0.05; 0.06]	.781
Political orientation x Politician x Country: Germany	−0.05	0.03	[-0.11; 0.02]	.181
Political orientation x Politician x Country: Israel	0.05	0.03	[-0.00; 0.10]	.057
Political orientation x Politician x Country: Luxembourg	−0.07	0.08	[-0.23; 0.09]	.392
Political orientation x Politician x Country: Netherlands	−0.09	0.06	[-0.20; 0.02]	.118
Political orientation x Politician x Country: Norway	0.08	0.04	[-0.00; 0.17]	.060
Education level x Politician x Country: Australia	0.01	0.12	[-0.22; 0.25]	.910
Education level x Politician x Country: Czechia	0.01	0.08	[-0.16; 0.17]	.931
Education level x Politician x Country: Flanders	0.07	0.06	[-0.05; 0.20]	.261
Education level x Politician x Country: Germany	0.12	0.07	[-0.02; 0.25]	.095
Education level x Politician x Country: Israel	−0.15	0.09	[-0.33; 0.03]	.111

(*Continued*) Results of unstandardized regression model on belief in climate change moderated by groups and countries

	Belief in climate change			
Predictors	*b*	*SE*[a]	*95% CI*	*p*
Education level x Politician x Country: Luxembourg	−0.01	0.11	[-0.22; 0.20]	.927
Education level x Politician x Country: Netherlands	−0.06	0.12	[-0.29; 0.18]	.645
Education level x Politician x Country: Norway	−0.11	0.10	[-0.30; 0.08]	.256
Political orientation x Education level x Country: Australia	0.04	0.02	[0.01; 0.08]	.016
Political orientation x Education level x Country: Czechia	−0.02	0.02	[-0.07; 0.02]	.293
Political orientation x Education level x Country: Flanders	−0.03	0.02	[-0.06; 0.00]	.062
Political orientation x Education level x Country: Germany	−0.01	0.02	[-0.05; 0.03]	.581
Political orientation x Education level x Country: Israel	−0.01	0.01	[-0.03; 0.02]	.603
Political orientation x Education level x Country: Luxembourg	−0.01	0.02	[-0.05; 0.02]	.499
Political orientation x Education level x Country: Netherlands	0.03	0.03	[-0.02; 0.08]	.259
Political orientation x Education level x Country: Norway	0.01	0.01	[-0.02; 0.03]	.600
df	18,932			
F	21.72***			
Multiple R^2 / *Adjusted* R^2	0.066 / 0.063			

Note. N = 18,995; b = Unstandardized regression coefficient; SE = Standard error; *95% CI* = 95% Confidence interval; p = p-value with a significance level of 5%.

[a]Robust standard errors were calculated with heteroscedasticity-consistent covariance matrix for the model. [b]effect-coded variable with -1 = Citizens and 1 = Politicians. [c]effect-coded variable with -1 = Lower education and 1 = Higher education. [d]grand-mean centered score with higher values indicating more right-leaning political orientation. [e]effect-coded variable with Wallonia as the reference level.

*** indicates $p < .001$

Supplementary Table S7

Table S7. Results of standardized regression model on belief in climate change moderated by groups and countries

	Belief in climate change			
Predictors	*β*	*SE*[a]	*95% CI*	*p*
Main effects				
Intercept	0.08	0.03	[0.02; 0.13]	.004
Politician[b]	0.06	0.03	[0.01; 0.11]	.024
Education level[c]	0.05	0.02	[0.01; 0.10]	.009
Political orientation[d]	−0.35	0.02	[-0.40; -0.31]	<.001
Education level x Politician	−0.01	0.02	[-0.06; 0.03]	.483
Political orientation x Politician	−0.18	0.02	[-0.22; -0.13]	<.001
Political orientation x Education level	−0.01	0.01	[-0.03; 0.01]	.529
Covariates				
Country: Australia[e]	−0.13	0.08	[-0.29; 0.03]	.115
Country: Czechia	0.03	0.06	[-0.09; 0.15]	.569
Country: Flanders	0.02	0.04	[-0.06; 0.11]	.610
Country: Germany	−0.13	0.06	[-0.24; -0.01]	.027
Country: Israel	0.12	0.06	[0.02; 0.23]	.026
Country: Luxembourg	−0.12	0.13	[-0.37; 0.13]	.346
Country: Netherlands	−0.14	0.11	[-0.35; 0.07]	.195
Country: Norway	0.15	0.06	[0.02; 0.28]	.019
Politician x Country: Australia	−0.09	0.08	[-0.25; 0.07]	.250
Politician x Country: Czechia	0.12	0.06	[-0.00; 0.24]	.050
Politician x Country: Flanders	−0.10	0.04	[-0.19; -0.02]	.014
Politician x Country: Germany	−0.03	0.06	[-0.15; 0.08]	.565
Politician x Country: Israel	0.05	0.06	[-0.05; 0.16]	.332
Politician x Country: Luxembourg	−0.20	0.13	[-0.45; 0.04]	.108
Politician x Country: Netherlands	−0.11	0.11	[-0.32; 0.10]	.310
Politician x Country: Norway	0.28	0.06	[0.15; 0.40]	<.001
Education level x Country: Australia	0.10	0.08	[-0.05; 0.25]	.202
Education level x Country: Czechia	−0.10	0.05	[-0.20; 0.01]	.068
Education level x Country: Flanders	0.03	0.04	[-0.04; 0.11]	.396
Education level x Country: Germany	0.02	0.05	[-0.07; 0.12]	.627
Education level x Country: Israel	−0.15	0.06	[-0.27; -0.04]	.009
Education level x Country: Luxembourg	0.07	0.07	[-0.06; 0.21]	.291
Education level x Country: Netherlands	−0.06	0.07	[-0.21; 0.08]	.381
Education level x Country: Norway	0.07	0.06	[-0.05; 0.19]	.229
Political orientation x Country: Australia	−0.10	0.07	[-0.23; 0.04]	.172
Political orientation x Country: Czechia	0.14	0.06	[0.01; 0.26]	.031
Political orientation x Country: Flanders	0.00	0.04	[-0.07; 0.08]	.945
Political orientation x Country: Germany	−0.12	0.05	[-0.21; -0.03]	.012
Political orientation x Country: Israel	0.08	0.04	[0.01; 0.16]	.032
Political orientation x Country: Luxembourg	−0.14	0.12	[-0.38; 0.10]	.260
Political orientation x Country: Netherlands	−0.10	0.08	[-0.26; 0.05]	.195
Political orientation x Country: Norway	0.05	0.06	[-0.08; 0.17]	.464
Political orientation x Politician x Country: Australia	−0.05	0.07	[-0.19; 0.09]	.503
Political orientation x Politician x Country: Czechia	0.02	0.07	[-0.11; 0.16]	.713
Political orientation x Politician x Country: Flanders	0.01	0.04	[-0.07; 0.09]	.781
Political orientation x Politician x Country: Germany	−0.07	0.05	[-0.17; 0.03]	.181
Political orientation x Politician x Country: Israel	0.08	0.04	[-0.00; 0.15]	.057
Political orientation x Politician x Country: Luxembourg	−0.11	0.12	[-0.35; 0.14]	.392
Political orientation x Politician x Country: Netherlands	−0.13	0.08	[-0.30; 0.03]	.118
Political orientation x Politician x Country: Norway	0.12	0.06	[-0.01; 0.25]	.060
Education level x Politician x Country: Australia	0.01	0.08	[-0.14; 0.16]	.910
Education level x Politician x Country: Czechia	0.00	0.05	[-0.10; 0.11]	.931
Education level x Politician x Country: Flanders	0.05	0.04	[-0.03; 0.13]	.261
Education level x Politician x Country: Germany	0.07	0.04	[-0.01; 0.16]	.095
Education level x Politician x Country: Israel	−0.09	0.06	[-0.21; 0.02]	.111

(*Continued*) Results of standardized regression model on belief in climate change moderated by groups and countries

	Belief in climate change			
Predictors	β	SE^{a}	*95% CI*	p
Education level x Politician x Country: Luxembourg	−0.01	0.07	[-0.14; 0.12]	.927
Education level x Politician x Country: Netherlands	−0.03	0.08	[-0.18; 0.11]	.645
Education level x Politician x Country: Norway	−0.07	0.06	[-0.19; 0.05]	.256
Political orientation x Education level x Country: Australia	0.07	0.03	[0.01; 0.12]	.016
Political orientation x Education level x Country: Czechia	−0.03	0.03	[-0.10; 0.03]	.293
Political orientation x Education level x Country: Flanders	−0.05	0.02	[-0.10; 0.00]	.062
Political orientation x Education level x Country: Germany	−0.02	0.03	[-0.08; 0.04]	.581
Political orientation x Education level x Country: Israel	−0.01	0.02	[-0.05; 0.03]	.603
Political orientation x Education level x Country: Luxembourg	−0.02	0.03	[-0.07; 0.03]	.499
Political orientation x Education level x Country: Netherlands	0.04	0.04	[-0.03; 0.12]	.259
Political orientation x Education level x Country: Norway	0.01	0.02	[-0.03; 0.05]	.600
df	18,932			
F	21.72***			
Multiple R^2 / *Adjusted* R^2	0.066 / 0.063			

Note. N = 18,995; β = Standardized regression coefficient; *SE* = Standard error; *95% CI* = 95% Confidence interval; p = p-value with a significance level of 5%.

[a]Robust standard errors were calculated with heteroscedasticity-consistent covariance matrix for the model. [b]effect-coded variable with -1 = Citizens and 1 = Politicians. [c]effect-coded variable with -1 = Lower education and 1 = Higher education. [d]z-standardized score with higher values indicating more right-leaning political orientation. [e]effect-coded variable with Wallonia as the reference level.

*** indicates $p < .001$

Supplementary Table S8

Table S8. Overview of slopes of political orientation effect on belief in climate change per country in unstandardized regression

	Belief in climate change		
Country: Political orientation effect	*b*	*SE*[a]	*95% CI*
Australia	−0.30	0.05	[-0.40; -0.20]
Czechia	−0.15	0.04	[-0.23; -0.06]
Flanders	−0.23	0.02	[-0.28; -0.19]
Germany	−0.32	0.03	[-0.38; -0.26]
Israel	−0.18	0.02	[-0.23; -0.14]
Luxembourg	−0.33	0.09	[-0.51; -0.15]
Netherlands	−0.31	0.06	[-0.42; -0.19]
Norway	−0.20	0.05	[-0.29; -0.12]
Wallonia	−0.11	0.02	[-0.15; -0.07]
df	18,932		

Note. N = 18,995; b = Unstandardized regression coefficient; *SE* = Standard error; *95% CI* = 95% Confidence interval.
[a]Robust standard errors were calculated with heteroscedasticity-consistent covariance matrix for the model.

Supplementary Table S9

Table S9. Estimated marginal means and contrasts at ±1 standard deviation of political orientation for politicians and citizens

	Belief in climate change in politicians			
Political orientation	Estimated marginal mean	*SE*[a]	df	*95% CI*
Left (2.86)[b]	6.21	0.07	18,932	[6.07; 6.35]
Mean (5.23)	5.37	0.08	18,932	[5.20; 5.54]
Right (7.6)	4.53	0.15	18,932	[4.25; 4.81]

	Belief in climate change in citizens			
Political orientation	Estimated marginal mean	*SE*[a]	df	*95% CI*
Left (2.86)[b]	5.46	0.02	18,932	[5.41; 5.50]
Mean (5.23)	5.17	0.02	18,932	[5.14; 5.20]
Right (7.6)	4.89	0.02	18,932	[4.85; 4.94]

	Difference in belief in climate change				
Contrast	Estimate	*SE*[a]	df	*t*	*p*
Left politicians vs. left citizens	0.752	0.07	18,932	10.157	<.001
Right politicians vs. right citizens	-0.362	0.15	18,932	-2.460	.014
Left politicians vs. right politicians	1.316	0.07	18,932	17.949	<.001
Left citizens vs. right citizens	0.564	0.03	18,932	17.307	<.001

Note. $N_{\text{Politicians}} = 714$; $N_{\text{Citizens}} = 18{,}281$; *SE* = Standard error; p = p-value with a significance level of 5%. Estimated marginal means of belief in climate change for politicians and citizens and contrasts based on the unstandardized regression model with robust standard errors.

[a]Robust standard errors were calculated with heteroscedasticity-consistent covariance matrix for the model. [b]Marginal means were estimated at ±1 standard deviation from the full sample mean of political orientation (5.23 on a scale from '0' = left to '10' = right).

Supplementary Table S10

Table S10. Results of Type-III ANOVA for unstandardized regression model on belief in climate change moderated by groups and countries

	Belief in climate change		
Predictors	*F*	*df*	*p*
Intercept	14 943.89	1,18932	<.001
Politician	5.11	1,18932	.024
Education level	6.80	1,18932	.009
Political orientation	217.03	1,18932	<.001
Country	3.74	8,18932	<.001
Politician x Country	4.77	8,18932	<.001
Political orientation x Politician	50.67	1,18932	<.001
Political orientation x Country	5.80	8,18932	<.001
Education level x Politician	0.49	1,18932	.483
Education level x Country	1.83	8,18932	.067
Political orientation x Education level	0.40	1,18932	.529
Political orientation x Politician x Country	2.35	8,18932	.016
Education level x Politician x Country	1.15	8,18932	.323
Political orientation x Education level x Country	1.46	8,18932	.167
df(Residuals)	18,932		

Note. N = 18,995; p = p-value with a significance level of 5%.

Supplementary Table S11

Table S11. Overview of slopes of unstandardized political orientation effect on belief in climate change per group for each country

	Belief in climate change		
Country: Political orientation effect	*b*	*SE*[a]	*95% CI*
Australia			
Politicians	−0.45	0.10	[-0.64; -0.26]
Citizens	−0.15	0.02	[-0.19; -0.11]
Czechia			
Politicians	−0.25	0.09	[-0.42; -0.07]
Citizens	−0.04	0.02	[-0.09; 0.00]
Flanders			
Politicians	−0.34	0.05	[-0.43; -0.26]
Citizens	−0.12	0.02	[-0.16; -0.09]
Germany			
Politicians	−0.48	0.06	[-0.60; -0.36]
Citizens	−0.15	0.02	[-0.20; -0.11]
Israel			
Politicians	−0.25	0.05	[-0.34; -0.16]
Citizens	−0.11	0.01	[-0.14; -0.09]
Luxembourg			
Politicians	−0.51	0.18	[-0.87; -0.16]
Citizens	−0.10	0.02	[-0.18; -0.10]
Netherlands			
Politicians	−0.51	0.12	[-0.74; -0.28]
Citizens	−0.10	0.03	[-0.16; -0.04]
Norway			
Politicians	−0.24	0.09	[-0.42; -0.06]
Citizens	−0.17	0.01	[-0.20; -0.14]
Wallonia			
Politicians	−0.15	0.04	[-0.22; -0.07]
Citizens	−0.07	0.02	[-0.12; -0.03]
df	18,932		

Note. N = 18,995; b = Unstandardized regression coefficient; *SE* = Standard error; *95% CI* = 95% Confidence interval.
[a]Robust standard errors were calculated with heteroscedasticity-consistent covariance matrix for the model.

Supplementary Table S12

Table S12. Contrasts of unstandardized political orientation effect on belief in climate change between groups for each country

	Belief in climate change			
Contrast for political orientation effect	Estimate[a]	SE[b]	t	p
Australia: Politicans vs. Citizens	−0.30	0.10	−2.965	.003
Czechia: Politicans vs. Citizens	−0.20	0.09	−2.127	.034
Flanders: Politicans vs. Citizens	−0.22	0.05	−4.358	<.001
Germany: Politicans vs. Citizens	−0.33	0.07	−4.739	<.001
Israel: Politicans vs. Citizens	−0.13	0.05	−2.825	.005
Luxembourg: Politicans vs. Citizens	−0.38	0.18	−2.049	.040
Netherlands: Politicans vs. Citizens	−0.41	0.12	−3.346	<.001
Norway: Politicans vs. Citizens	−0.07	0.09	−0.777	.437
Wallonia: Politicans vs. Citizens	−0.07	0.05	−1.552	.121
df	18,932			

Note. $N = 18{,}995$; SE = Standard error; p = p-value with a significance level of 5%.
[a]Difference in the effect of political orientation on belief in climate change between groups with negative values indicating stronger negative effect in politicians.
[b]Robust standard errors were calculated with heteroscedasticity-consistent covariance matrix for the model.

Supplementary Table S13

Table S13. Results of unstandardized regression model on belief in climate change moderated by group with gender as control variable

	Belief in climate change			
Predictors	*b*	*SE*[a]	*95% CI*	*p*
Main effects				
Intercept	5.23	0.05	[5.13; 5.32]	<.001
Politician[b]	0.05	0.05	[-0.05; 0.14]	.319
Gender[c]	0.06	0.01	[0.04; 0.08]	<.001
Education level[d]	0.13	0.04	[0.06; 0.20]	<.001
Political orientation[e]	−0.24	0.02	[-0.27; -0.21]	<.001
Education level x Politician	0.01	0.04	[-0.06; 0.09]	.688
Political orientation x Politician	−0.12	0.02	[-0.16; -0.09]	<.001
Political orientation x Education level	−0.01	0.01	[-0.02; 0.01]	.398
Covariates				
Country: Australia[f]	−0.22	0.14	[-0.50; 0.06]	.116
Country: Czechia	0.14	0.10	[-0.06; 0.33]	.181
Country: Flanders	0.08	0.07	[-0.07; 0.23]	.294
Country: Germany	−0.14	0.10	[-0.32; 0.05]	.150
Country: Israel	0.28	0.09	[0.10; 0.46]	.003
Country: Luxembourg	−0.54	0.26	[-1.04; -0.03]	.039
Country: Netherlands	−0.17	0.17	[-0.51; 0.17]	.335
Country: Norway	0.24	0.11	[0.03; 0.46]	.028
Politician x Country: Australia	−0.17	0.14	[-0.44; 0.11]	.242
Politician x Country: Czechia	0.26	0.10	[0.06; 0.46]	.010
Politician x Country: Flanders	−0.12	0.07	[-0.26; 0.02]	.111
Politician x Country: Germany	0.01	0.10	[-0.18; 0.20]	.892
Politician x Country: Israel	0.17	0.09	[-0.01; 0.36]	.060
Politician x Country: Luxembourg	−0.67	0.26	[-1.18; -0.16]	.010
Politician x Country: Netherlands	−0.12	0.17	[-0.46; 0.21]	.475
Politician x Country: Norway	0.45	0.11	[0.23; 0.67]	<.001
Education level x Country: Australia	0.17	0.13	[-0.09; 0.42]	.200
Education level x Country: Czechia	−0.19	0.09	[-0.36; -0.02]	.031
Education level x Country: Flanders	0.03	0.07	[-0.11; 0.17]	.685
Education level x Country: Germany	−0.01	0.08	[-0.16; 0.14]	.885
Education level x Country: Israel	−0.25	0.10	[-0.44; -0.06]	.009
Education level x Country: Luxembourg	0.32	0.14	[0.05; 0.59]	.022
Education level x Country: Netherlands	−0.13	0.12	[-0.36; 0.10]	.279
Education level x Country: Norway	0.07	0.11	[-0.14; 0.27]	.537
Political orientation x Country: Australia	−0.05	0.05	[-0.14; 0.04]	.278
Political orientation x Country: Czechia	0.09	0.04	[-0.00; 0.17]	.052
Political orientation x Country: Flanders	0.02	0.03	[-0.03; 0.08]	.429
Political orientation x Country: Germany	−0.07	0.03	[-0.13; -0.01]	.032
Political orientation x Country: Israel	0.07	0.03	[0.01; 0.12]	.016
Political orientation x Country: Luxembourg	−0.17	0.09	[-0.34; 0.01]	.064
Political orientation x Country: Netherlands	−0.06	0.05	[-0.16; 0.05]	.290
Political orientation x Country: Norway	0.04	0.05	[-0.06; 0.13]	.449
Political orientation x Politician x Country: Australia	−0.02	0.05	[-0.11; 0.08]	.699
Political orientation x Politician x Country: Czechia	0.01	0.05	[-0.08; 0.10]	.777
Political orientation x Politician x Country: Flanders	0.03	0.03	[-0.03; 0.08]	.364
Political orientation x Politician x Country: Germany	−0.04	0.04	[-0.11; 0.03]	.283
Political orientation x Politician x Country: Israel	0.06	0.03	[0.01; 0.12]	.029
Political orientation x Politician x Country: Luxembourg	−0.15	0.09	[-0.32; 0.03]	.101
Political orientation x Politician x Country: Netherlands	−0.07	0.06	[-0.19; 0.04]	.201
Political orientation x Politician x Country: Norway	0.08	0.05	[-0.01; 0.18]	.072
Education level x Politician x Country: Australia	0.02	0.13	[-0.24; 0.28]	.878
Education level x Politician x Country: Czechia	−0.03	0.09	[-0.20; 0.14]	.725
Education level x Politician x Country: Flanders	0.05	0.07	[-0.09; 0.19]	.498
Education level x Politician x Country: Germany	0.07	0.07	[-0.08; 0.21]	.362

Predictors	Belief in climate change *b*	*SE*[a]	*95% CI*	*p*
Education level x Politician x Country: Israel	−0.15	0.10	[-0.34; 0.04]	.127
Education level x Politician x Country: Luxembourg	0.20	0.13	[-0.07; 0.46]	.145
Education level x Politician x Country: Netherlands	−0.09	0.12	[-0.33; 0.15]	.450
Education level x Politician x Country: Norway	−0.15	0.11	[-0.36; 0.05]	.147
Political orientation x Education level x Country: Australia	0.05	0.02	[0.01; 0.08]	.012
Political orientation x Education level x Country: Czechia	−0.03	0.02	[-0.07; 0.02]	.227
Political orientation x Education level x Country: Flanders	−0.04	0.02	[-0.07; -0.00]	.035
Political orientation x Education level x Country: Germany	−0.01	0.02	[-0.05; 0.03]	.668
Political orientation x Education level x Country: Israel	−0.01	0.01	[-0.03; 0.02]	.665
Political orientation x Education level x Country: Luxembourg	−0.01	0.02	[-0.04; 0.03]	.635
Political orientation x Education level x Country: Netherlands	0.03	0.03	[-0.02; 0.08]	.316
Political orientation x Education level x Country: Norway	0.01	0.01	[-0.02; 0.04]	.516
df	18,792			
F	20.82***			
Multiple R^2 / *Adjusted* R^2	0.066 / 0.063			

Note. N = 18,857; b = Unstandardized regression coefficient; *SE* = Standard error; *95% CI* = 95% Confidence interval; p = p-value with a significance level of 5%.
[a]Robust standard errors were calculated with heteroscedasticity-consistent covariance matrix for the model. [b]effect-coded variable with -1 = Citizens and 1 = Politicians. [c]effect-coded variable with -1 = Male and 1 = Female. [d]effect-coded variable with -1 = Lower education and 1 = Higher education. [e]grand-mean centered score with higher values indicating more right-leaning political orientation. [f]effect-coded variable with Wallonia as the reference level.
*** indicates $p < .001$

Supplementary Table S14

Table S14. Results of standardized regression model on belief in climate change moderated by group with gender as control variable

	Belief in climate change			
Predictors	β	SE^a	*95% CI*	*p*
Main effects				
Intercept	0.05	0.03	[-0.01; 0.11]	.112
Politician[b]	0.03	0.03	[-0.03; 0.09]	.319
Gender[c]	0.04	0.01	[0.02; 0.05]	<.001
Education level[d]	0.08	0.02	[0.04; 0.12]	<.001
Political orientation[e]	−0.36	0.03	[-0.41; -0.31]	<.001
Education level x Politician	0.01	0.02	[-0.04; 0.05]	.688
Political orientation x Politician	−0.18	0.03	[-0.24; -0.13]	<.001
Political orientation x Education level	−0.01	0.01	[-0.03; 0.01]	.398
Covariates				
Country: Australia[f]	−0.14	0.09	[-0.32; 0.03]	.116
Country: Czechia	0.09	0.06	[-0.04; 0.21]	.181
Country: Flanders	0.05	0.05	[-0.04; 0.14]	.294
Country: Germany	−0.09	0.06	[-0.20; 0.03]	.150
Country: Israel	0.18	0.06	[0.06; 0.29]	.003
Country: Luxembourg	−0.34	0.16	[-0.66; -0.02]	.039
Country: Netherlands	−0.10	0.11	[-0.32; 0.11]	.335
Country: Norway	0.15	0.07	[0.02; 0.29]	.028
Politician x Country: Australia	−0.10	0.09	[-0.28; 0.07]	.242
Politician x Country: Czechia	0.16	0.06	[0.04; 0.29]	.010
Politician x Country: Flanders	−0.07	0.05	[-0.17; 0.02]	.111
Politician x Country: Germany	0.01	0.06	[-0.11; 0.13]	.892
Politician x Country: Israel	0.11	0.06	[-0.00; 0.22]	.060
Politician x Country: Luxembourg	−0.42	0.16	[-0.74; -0.10]	.010
Politician x Country: Netherlands	−0.08	0.11	[-0.29; 0.13]	.475
Politician x Country: Norway	0.28	0.07	[0.15; 0.42]	<.001
Education level x Country: Australia	0.11	0.08	[-0.06; 0.27]	.200
Education level x Country: Czechia	−0.12	0.05	[-0.22; -0.01]	.031
Education level x Country: Flanders	0.02	0.04	[-0.07; 0.10]	.685
Education level x Country: Germany	−0.01	0.05	[-0.10; 0.09]	.885
Education level x Country: Israel	−0.16	0.06	[-0.28; -0.04]	.009
Education level x Country: Luxembourg	0.20	0.09	[0.03; 0.37]	.022
Education level x Country: Netherlands	−0.08	0.08	[-0.23; 0.07]	.279
Education level x Country: Norway	0.04	0.07	[-0.09; 0.17]	.537
Political orientation x Country: Australia	−0.08	0.07	[-0.21; 0.06]	.278
Political orientation x Country: Czechia	0.13	0.07	[-0.00; 0.25]	.052
Political orientation x Country: Flanders	0.03	0.04	[-0.05; 0.11]	.429
Political orientation x Country: Germany	−0.11	0.05	[-0.20; -0.01]	.032
Political orientation x Country: Israel	0.10	0.04	[0.02; 0.18]	.016
Political orientation x Country: Luxembourg	−0.25	0.13	[-0.51; 0.01]	.064
Political orientation x Country: Netherlands	−0.09	0.08	[-0.24; 0.07]	.290
Political orientation x Country: Norway	0.05	0.07	[-0.08; 0.19]	.449
Political orientation x Politician x Country: Australia	−0.03	0.07	[-0.17; 0.11]	.699
Political orientation x Politician x Country: Czechia	0.02	0.07	[-0.12; 0.16]	.777
Political orientation x Politician x Country: Flanders	0.04	0.04	[-0.05; 0.13]	.364
Political orientation x Politician x Country: Germany	−0.06	0.05	[-0.16; 0.05]	.283
Political orientation x Politician x Country: Israel	0.09	0.04	[0.01; 0.18]	.029
Political orientation x Politician x Country: Luxembourg	−0.22	0.13	[-0.48; 0.04]	.101
Political orientation x Politician x Country: Netherlands	−0.11	0.09	[-0.28; 0.06]	.201
Political orientation x Politician x Country: Norway	0.13	0.07	[-0.01; 0.26]	.072
Education level x Politician x Country: Australia	0.01	0.08	[-0.15; 0.17]	.878
Education level x Politician x Country: Czechia	−0.02	0.05	[-0.12; 0.09]	.725
Education level x Politician x Country: Flanders	0.03	0.04	[-0.06; 0.12]	.498
Education level x Politician x Country: Germany	0.04	0.05	[-0.05; 0.13]	.362

	Belief in climate change			
Predictors	β	SE[a]	*95% CI*	p
Education level x Politician x Country: Israel	−0.09	0.06	[-0.21; 0.03]	.127
Education level x Politician x Country: Luxembourg	0.12	0.08	[-0.04; 0.29]	.145
Education level x Politician x Country: Netherlands	−0.06	0.08	[-0.21; 0.09]	.450
Education level x Politician x Country: Norway	−0.10	0.07	[-0.23; 0.03]	.147
Political orientation x Education level x Country: Australia	0.07	0.03	[0.02; 0.12]	.012
Political orientation x Education level x Country: Czechia	−0.04	0.03	[-0.11; 0.02]	.227
Political orientation x Education level x Country: Flanders	−0.05	0.03	[-0.10; -0.00]	.035
Political orientation x Education level x Country: Germany	−0.01	0.03	[-0.07; 0.05]	.668
Political orientation x Education level x Country: Israel	−0.01	0.02	[-0.04; 0.03]	.665
Political orientation x Education level x Country: Luxembourg	−0.01	0.03	[-0.06; 0.04]	.635
Political orientation x Education level x Country: Netherlands	0.04	0.04	[-0.04; 0.11]	.316
Political orientation x Education level x Country: Norway	0.01	0.02	[-0.03; 0.05]	.516
df	18,792			
F	20.82***			
Multiple R^2 / *Adjusted* R^2	0.066 / 0.062			

Note. N = 18,857; β = Standardized regression coefficient; SE = Standard error; *95% CI* = 95% Confidence interval; p = p-value with a significance level of 5%.
[a]Robust standard errors were calculated with heteroscedasticity-consistent covariance matrix for the model. [b]effect-coded variable with -1 = Citizens and 1 = Politicians. [c]effect-coded variable with -1 = Male and 1 = Female. [d]effect-coded variable with -1 = Lower education and 1 = Higher education. [e]z-standardized score with higher values indicating more right-leaning political orientation. [f]effect-coded variable with Wallonia as the reference level.
*** indicates $p < .001$

Supplementary Table S15

Table S15. Results of unstandardized regression model on belief in climate change among citizens with age as control variable

Predictors	Belief in climate change			
	b	*SE*	*95% CI*	*p*
Main effects				
Intercept	5.36	0.04	[5.29; 5.43]	<.001
Age	0.00	0.00	[-0.01; -0.00]	<.001
Education level[a]	0.10	0.02	[0.07; 0.13]	<.001
Political orientation[b]	−0.12	0.01	[-0.13; -0.11]	<.001
Political orientation x Education	0.00	0.01	[-0.02; 0.01]	.515
Covariates				
Country: Australia[c]	−0.07	0.04	[-0.15; 0.01]	.105
Country: Czechia	−0.13	0.04	[-0.21; -0.04]	.003
Country: Flanders	0.20	0.04	[0.12; 0.27]	<.001
Country: Germany	−0.14	0.04	[-0.22; -0.06]	.001
Country: Israel	0.10	0.03	[0.04; 0.16]	.001
Country: Luxembourg	0.14	0.04	[0.07; 0.21]	<.001
Country: Netherlands	−0.04	0.06	[-0.16; 0.08]	.543
Country: Norway	−0.20	0.04	[-0.27; -0.13]	<.001
Education level x Country: Australia	0.13	0.04	[0.04; 0.21]	.003
Education level x Country: Czechia	−0.15	0.04	[-0.24; -0.07]	<.001
Education level x Country: Flanders	−0.02	0.04	[-0.10; 0.05]	.595
Education level x Country: Germany	−0.07	0.04	[-0.16; 0.01]	.079
Education level x Country: Israel	−0.09	0.03	[-0.15; -0.03]	.003
Education level x Country: Luxembourg	0.12	0.04	[0.05; 0.20]	.001
Education level x Country: Netherlands	−0.05	0.06	[-0.17; 0.07]	.397
Education level x Country: Norway	0.23	0.04	[0.15; 0.30]	<.001
Political orientation x Country: Australia	−0.03	0.02	[-0.07; 0.01]	.091
Political orientation x Country: Czechia	0.07	0.02	[0.03; 0.11]	.002
Political orientation x Country: Flanders	0.00	0.02	[-0.04; 0.03]	.944
Political orientation x Country: Germany	−0.03	0.02	[-0.08; 0.01]	.111
Political orientation x Country: Israel	0.00	0.01	[-0.02; 0.03]	.942
Political orientation x Country: Luxembourg	−0.02	0.02	[-0.05; 0.02]	.367
Political orientation x Country: Netherlands	0.01	0.03	[-0.04; 0.07]	.633
Political orientation x Country: Norway	−0.05	0.01	[-0.08; -0.02]	<.001
Political orientation x Education level x Country: Australia	0.04	0.02	[0.01; 0.08]	.017
Political orientation x Education level x Country: Czechia	−0.03	0.02	[-0.07; 0.02]	.255
Political orientation x Education level x Country: Flanders	−0.03	0.02	[-0.06; 0.01]	.097
Political orientation x Education level x Country: Germany	−0.01	0.02	[-0.06; 0.03]	.496
Political orientation x Education level x Country: Israel	0.00	0.01	[-0.03; 0.02]	.771
Political orientation x Education level x Country: Luxembourg	−0.01	0.02	[-0.05; 0.02]	.544
Political orientation x Education level x Country: Netherlands	0.03	0.03	[-0.03; 0.08]	.338
Political orientation x Education level x Country: Norway	0.01	0.01	[-0.02; 0.03]	.647
df	18,175			
F	29.01***			
Multiple R^2 / *Adjusted* R^2	0.054 / 0.052			

Note. N = 18,212; b = Unstandardized regression coefficient; *SE* = Standard error; *95% CI* = 95% Confidence interval; p = p-value with a significance level of 5%.
[a]effect-coded variable with -1 = Lower education and 1 = Higher education. [b]grand-mean centered score with higher values indicating more right-leaning political orientation. [c]effect-coded variable with Wallonia as the reference level.
*** indicates $p < .001$

Supplementary Table S16

Table S16. Results of standardized regression model on belief in climate change among citizens with age as control variable

	Belief in climate change			
Predictors	*β*	*SE*	*95% CI*	*p*
Main effects				
Intercept	0.03	0.01	[0.01; 0.04]	.009
Age	−0.04	0.01	[-0.06; -0.03]	<.001
Education level[a]	0.06	0.01	[0.04; 0.08]	<.001
Political orientation[b]	−0.18	0.01	[-0.20; -0.16]	<.001
Political orientation x Education	−0.01	0.01	[-0.03; 0.01]	.515
Covariates				
Country: Australia[c]	−0.04	0.03	[-0.10; 0.01]	.105
Country: Czechia	−0.08	0.03	[-0.13; -0.03]	.003
Country: Flanders	0.12	0.02	[0.08; 0.17]	<.001
Country: Germany	−0.09	0.03	[-0.14; -0.04]	.001
Country: Israel	0.06	0.02	[0.03; 0.10]	.001
Country: Luxembourg	0.09	0.02	[0.04; 0.13]	<.001
Country: Netherlands	−0.02	0.04	[-0.10; 0.05]	.543
Country: Norway	−0.13	0.02	[-0.17; -0.08]	<.001
Education level x Country: Australia	0.08	0.03	[0.03; 0.13]	.003
Education level x Country: Czechia	−0.10	0.03	[-0.15; -0.04]	<.001
Education level x Country: Flanders	−0.01	0.02	[-0.06; 0.03]	.595
Education level x Country: Germany	−0.05	0.03	[-0.10; 0.01]	.079
Education level x Country: Israel	−0.06	0.02	[-0.10; -0.02]	.003
Education level x Country: Luxembourg	0.08	0.02	[0.03; 0.12]	.001
Education level x Country: Netherlands	−0.03	0.04	[-0.11; 0.04]	.397
Education level x Country: Norway	0.14	0.02	[0.10; 0.19]	<.001
Political orientation x Country: Australia	−0.05	0.03	[-0.10; 0.01]	.091
Political orientation x Country: Czechia	0.10	0.03	[0.04; 0.17]	.002
Political orientation x Country: Flanders	0.00	0.03	[-0.05; 0.05]	.944
Political orientation x Country: Germany	−0.05	0.03	[-0.11; 0.01]	.111
Political orientation x Country: Israel	0.00	0.02	[-0.04; 0.04]	.942
Political orientation x Country: Luxembourg	−0.02	0.03	[-0.08; 0.03]	.367
Political orientation x Country: Netherlands	0.02	0.04	[-0.06; 0.10]	.633
Political orientation x Country: Norway	−0.07	0.02	[-0.12; -0.03]	<.001
Political orientation x Education level x Country: Australia	0.07	0.03	[0.01; 0.12]	.017
Political orientation x Education level x Country: Czechia	−0.04	0.03	[-0.10; 0.03]	.255
Political orientation x Education level x Country: Flanders	−0.04	0.03	[-0.10; 0.01]	.097
Political orientation x Education level x Country: Germany	−0.02	0.03	[-0.08; 0.04]	.496
Political orientation x Education level x Country: Israel	−0.01	0.02	[-0.04; 0.03]	.771
Political orientation x Education level x Country: Luxembourg	−0.02	0.03	[-0.07; 0.04]	.544
Political orientation x Education level x Country: Netherlands	0.04	0.04	[-0.04; 0.12]	.338
Political orientation x Education level x Country: Norway	0.01	0.02	[-0.03; 0.05]	.647
df	18,175			
F	29.01***			
Multiple R^2 / Adjusted R^2	0.054 / 0.052			

Note. N = 18,212; β = Standardized regression coefficient; *SE* = Standard error; *95% CI* = 95% Confidence interval; p = p-value with a significance level of 5%.
[a]effect-coded variable with -1 = Lower education and 1 = Higher education. [b]z-standardized score with higher values indicating more right-leaning political orientation. [c]effect-coded variable with Wallonia as the reference level.
*** indicates $p < .001$

Supplementary Table S17

Table S17. Results of unstandardized path analysis on policy support to increase airplane ticket prices with belief in climate change as mediator among politicians

Paths	*b*	*SE*	*99% CI*	*z*	*p*
Effects on belief in climate change					
Education[a]	0.27	0.12	[-0.04; 0.59]	2.223	.026
Political orientation[b]	−0.33	0.02	[-0.38; -0.27]	−16.228	<.001
Direct effects on policy support					
Education	0.17	0.14	[-0.19; 0.53]	1.208	.227
Political orientation	−0.10	0.03	[-0.17; -0.02]	−3.346	.001
Belief in climate change[c]	0.59	0.04	[0.47; 0.69]	13.615	<.001
Indirect effects on policy support					
Education	0.16	0.07	[-0.02; 0.36]	2.206	.027
Political orientation	−0.19	0.02	[-0.24; -0.14]	−10.253	<.001
Total effects on policy support					
Education	0.33	0.16	[-0.07; 0.72]	2.100	.037
Political orientation	−0.29	0.03	[-0.36; -0.22]	−10.767	<.001

Note. $N = 709$; b = Unstandardized regression coefficient; *SE* = Standard error; *99% CI* = 99% Bootstrapped Confidence interval; z = z-score; p = p-value with a significance level of 5%.
[a]effect-coded variable with -1 = Lower education and 1 = Higher education. [b]grand-mean centered score with higher values indicating more right-leaning political orientation. [c]raw score with higher values indicating higher belief in climate change.

Supplementary Table S18

Table S18. Results of standardized path analysis on policy support to increase airplane ticket prices with belief in climate change as mediator among politicians

Paths	β	*SE*	*99% CI*	*z*	*p*
Effects on belief in climate change					
Education[a]	0.18	0.08	[-0.03; 0.39]	2.223	.026
Political orientation[b]	−0.54	0.03	[-0.62; -0.45]	−16.228	<.001
Direct effects on policy support					
Education	0.09	0.07	[-0.10; 0.28]	1.208	.227
Political orientation	−0.13	0.04	[-0.23; -0.03]	−3.346	.001
Belief in climate change[c]	0.48	0.04	[0.38; 0.56]	13.615	<.001
Indirect effects on policy support					
Education	0.08	0.04	[-0.01; 0.19]	2.206	.027
Political orientation	−0.26	0.03	[-0.32; -0.19]	−10.253	<.001
Total effects on policy support					
Education	0.17	0.08	[-0.04; 0.38]	2.100	.036
Political orientation	−0.39	0.04	[-0.48; -0.29]	−10.767	<.001

Note. $N = 709$; β = Standardized regression coefficient; *SE* = Standard error; *99% CI* = 99% Bootstrapped Confidence interval; z = z-score; p = p-value with a significance level of 5%.
[a]effect-coded variable with -1 = Lower education and 1 = Higher education. [b]z-standardized score with higher values indicating more right-leaning political orientation.
[c]z-standardized score with higher values indicating higher belief in climate change.

Supplementary Table S19

Table S19. Results of unstandardized path analysis on policy support to subsidize the purchase of electric vehicles with belief in climate change as mediator among politicians

Paths	*b*	*SE*	*99% CI*	*z*	*p*
Effects on belief in climate change					
Education[a]	0.27	0.12	[-0.03; 0.59]	2.252	.024
Political orientation[b]	−0.33	0.02	[-0.38; -0.27]	−15.985	<.001
Direct effects on policy support					
Education	−0.13	0.15	[-0.51; 0.25]	−0.863	.388
Political orientation	−0.07	0.03	[-0.14; 0.003]	−2.492	.013
Belief in climate change[c]	0.32	0.04	[0.21; 0.43]	7.527	<.001
Indirect effects on policy support					
Education	0.09	0.04	[-0.01; 0.21]	2.180	.029
Political orientation	−0.11	0.02	[-0.15; -0.06]	−6.461	<.001
Total effects on policy support					
Education	−0.04	0.15	[-0.43; 0.35]	−0.253	.797
Political orientation	−0.18	0.02	[-0.24; -0.11]	−7.242	<.001

Note. N = 713; b = Unstandardized regression coefficient; *SE* = Standard error; *99% CI* = 99% Bootstrapped Confidence interval; z = z-score; p = p-value with a significance level of 5%.
[a]effect-coded variable with -1 = Lower education and 1 = Higher education. [b]grand-mean centered score with higher values indicating more right-leaning political orientation. [c]raw score with higher values indicating higher belief in climate change.

Supplementary Table S20

Table S20. Results of standardized path analysis on policy support to subsidize the purchase of electric vehicles with belief in climate change as mediator among politicians

Paths	β	*SE*	*99% CI*	z	p
Direct effects on belief in climate change					
Education[a]	0.18	0.08	[-0.02; 0.39]	2.252	.024
Political orientation[b]	−0.54	0.03	[-0.63; -0.45]	−15.982	<.001
Direct effects on policy support					
Education	−0.07	0.09	[-0.30; 0.15]	−0.863	.388
Political orientation	−0.11	0.04	[-0.21; 0.005]	−2.491	.013
Belief in climate change[c]	0.29	0.04	[0.19; 0.39]	7.527	<.001
Indirect effects on policy support					
Education	0.05	0.02	[-0.005; 0.12]	2.180	.029
Political orientation	−0.16	0.02	[-0.22; -0.10]	−6.461	<.001
Total effects on policy support					
Education	−0.02	0.09	[-0.25; 0.21]	−0.253	.800
Political orientation	−0.26	0.04	[-0.35; -0.17]	−7.241	<.001

Note. $N = 713$; β = Standardized regression coefficient; *SE* = Standard error; *99% CI* = 99% Bootstrapped Confidence interval; z = z-score; p = p-value with a significance level of 5%.
[a]effect-coded variable with -1 = Lower education and 1 = Higher education. [b]z-standardized score with higher values indicating more right-leaning political orientation. [c]z-standardized score with higher values indicating higher belief in climate change.

Supplementary Table S21

Table S21. Results of standardized path analysis on policy support to increase airplane ticket prices with age category and gender as predictors among politicians

Paths	β	*SE*	*99% CI*	z	p
Effects on belief in climate change					
Gender[a]	0.11	0.08	[-0.10; 0.31]	1.327	.185
Education[b]	0.12	0.10	[-0.12; 0.37]	1.259	.208
Age category[c]	−0.18	0.08	[-0.39; 0.04]	−2.239	.025
Political orientation[d]	−0.57	0.04	[-0.68; -0.47]	−14.247	<.001
Direct effects on policy support					
Gender	0.16	0.08	[-0.04; 0.36]	2.006	.045
Education	0.06	0.09	[-0.16; 0.29]	0.734	.463
Age category	0.10	0.07	[-0.09; 0.30]	1.402	.161
Political orientation	−0.18	0.05	[-0.30; -0.05]	−3.662	<.001
Belief in climate change[e]	0.43	0.04	[0.32; 0.54]	10.334	<.001
Indirect effects on policy support					
Gender	0.05	0.04	[-0.04; 0.14]	1.287	.198
Education	0.05	0.04	[-0.05; 0.17]	1.247	.213
Age category	−0.08	0.04	[-0.18; 0.01]	−2.165	.030
Political orientation	−0.25	0.03	[-0.32; -0.17]	−8.405	<.001
Total effects on policy support					
Gender	0.20	0.08	[-0.02; 0.42]	2.387	.017
Education	0.12	0.10	[-0.13; 0.37]	1.216	.224
Age category	0.03	0.08	[-0.19; 0.23]	0.313	.755
Political orientation	−0.42	0.04	[-0.53; -0.31]	−9.653	<.001

Note. $N = 489$; β = Standardized regression coefficient; *SE* = Standard error; *99% CI* = 99% Bootstrapped Confidence interval; z = z-score; p = p-value with a significance level of 5%.
[a]effect-coded variable with -1 = Male and 1 = Female. [b]effect-coded variable with -1 = Lower education and 1 = Higher education. [c]effect-coded variable for age category with -1 = below median and 1 = above median. [d]z-standardized score with higher values indicating more right-leaning political orientation. [e]z-standardized score with higher values indicating higher belief in climate change.

 Johannes Kotz, Helge Giese, Christian Breunig, Maj-Britt Sterba, Nathalie Brack, Patrick Dumont, Marija Taflaga, Javier Olivera, Lior Sheffer, Annika Werner, Anam Kuraishi, Wolfgang Gaissmaier

Supplementary Table S22

Table S22. Results of standardized path analysis on policy support to subsidize the purchase of electric vehicles with age category and gender as predictors among politicians

Paths	β	*SE*	*99% CI*	z	p
Effects on belief in climate change					
Gender[a]	0.11	0.08	[-0.09; 0.32]	1.437	.151
Education[b]	0.12	0.10	[-0.13; 0.38]	1.258	.208
Age category[c]	−0.19	0.08	[-0.40; 0.02]	−2.276	.023
Political orientation[d]	−0.57	0.04	[-0.68; -0.48]	−14.275	<.001
Direct effects on policy support					
Gender	0.08	0.09	[-0.15; 0.30]	0.867	.386
Education	−0.03	0.10	[-0.29; 0.24]	−0.257	.797
Age category	0.03	0.09	[-0.19; 0.25]	0.391	.696
Political orientation	−0.12	0.05	[-0.25; 0.01]	−2.419	.016
Belief in climate change[e]	0.29	0.04	[0.18; 0.41]	6.682	<.001
Indirect effects on policy support					
Gender	0.03	0.02	[-0.02; 0.10]	1.385	.166
Education	0.04	0.03	[-0.04; 0.12]	1.235	.217
Age category	−0.06	0.03	[-0.13; 0.004]	−2.110	.035
Political orientation	−0.17	0.03	[-0.25; -0.10]	−5.799	<.001
Total effects on policy support					
Gender	0.11	0.09	[-0.13; 0.34]	1.217	.224
Education	0.01	0.11	[-0.27; 0.28]	0.098	.922
Age category	−0.02	0.09	[-0.26; 0.20]	−0.245	.806
Political orientation	−0.29	0.04	[-0.40; -0.18]	−6.655	<.001

Note. $N = 493$; β = Standardized regression coefficient; *SE* = Standard error; *99% CI* = 99% Bootstrapped Confidence interval; z = z-score; p = p-value with a significance level of 5%.
[a]effect-coded variable with -1 = Male and 1 = Female. [b]effect-coded variable with -1 = Lower education and 1 = Higher education. [c]effect-coded variable for age category with -1 = below median and 1 = above median. [d]z-standardized score with higher values indicating more right-leaning political orientation. [e]z-standardized score with higher values indicating higher belief in climate change.

Supplementary Table S23

Table S23. Results of unstandardized path analysis on policy support to increase airplane ticket prices with belief in climate change as mediator among citizens

Paths	*b*	*SE*	*99% CI*	*z*	*p*
Effects on belief in climate change					
Education[a]	0.23	0.03	[0.17; 0.30]	8.762	<.001
Political orientation[b]	−0.12	0.01	[-0.13; -0.10]	−21.887	<.001
Direct effects on policy support					
Education	−0.06	0.03	[-0.14; 0.03]	−1.782	.075
Political orientation	−0.07	0.01	[-0.09; -0.05]	−10.886	<.001
Belief in climate change[c]	0.27	0.01	[0.24; 0.29]	28.400	<.001
Indirect effects on policy support					
Education	0.06	0.01	[0.04; 0.08]	8.339	<.001
Political orientation	−0.03	0.002	[-0.04; -0.03]	−17.295	<.001
Total effects on policy support					
Education	0.004	0.03	[-0.08; 0.09]	0.124	.901
Political orientation	−0.10	0.01	[-0.12; -0.08]	−15.424	<.001

Note. N = 17,696; b = Unstandardized regression coefficient; *SE* = Standard error; *99% CI* = 99% Bootstrapped Confidence interval; z = z-score; p = p-value with a significance level of 5%.
[a]effect-coded variable with -1 = Lower education and 1 = Higher education. [b]grand-mean centered score with higher values indicating more right-leaning political orientation. [c]raw score with higher values indicating higher belief in climate change.

Supplementary Table S24

Table S24. Results of standardized path analysis on policy support to increase airplane ticket prices with belief in climate change as mediator among citizens

Paths	β	*SE*	*99% CI*	z	p
Effects on belief in climate change					
Education[a]	0.15	0.02	[0.10; 0.19]	8.768	<.001
Political orientation[b]	−0.17	0.01	[-0.19; -0.15]	−21.895	<.001
Direct effects on policy support					
Education	−0.03	0.02	[-0.07; 0.01]	−1.782	.075
Political orientation	−0.09	0.01	[-0.11; -0.07]	−10.888	<.001
Belief in climate change[c]	0.22	0.01	[0.20; 0.24]	28.395	<.001
Indirect effects on policy support					
Education	0.03	0.004	[0.02; 0.04]	8.343	<.001
Political orientation	−0.04	0.002	[-0.04; -0.03]	−17.294	<.001
Total effects on policy support					
Education	0.002	0.02	[-0.04; 0.05]	0.124	.901
Political orientation	−0.12	0.01	[-0.15; -0.10]	−15.431	<.001

Note. N = 17,696; β = Standardized regression coefficient; *SE* = Standard error; *99% CI* = 99% Bootstrapped Confidence interval; z = z-score; p = p-value with a significance level of 5%.
[a]effect-coded variable with -1 = Lower education and 1 = Higher education. [b]z-standardized score with higher values indicating more right-leaning political orientation. [c]z-standardized score with higher values indicating higher belief in climate change.

Supplementary Table S25

Table S25. Results of unstandardized path analysis on policy support to subsidize the purchase of electric vehicles with belief in climate change as mediator among citizens

Paths	*b*	*SE*	*99% CI*	*z*	*p*
Effects on belief in climate change					
Education[a]	0.24	0.03	[0.17; 0.31]	8.805	<.001
Political orientation[b]	−0.11	0.01	[-0.13; -0.10]	−21.423	<.001
Direct effects on policy support					
Education	0.02	0.03	[-0.06; 0.11]	0.765	.444
Political orientation	−0.02	0.01	[-0.04; -0.01]	−3.683	<.001
Belief in climate change[c]	0.29	0.01	[0.27; 0.32]	30.684	<.001
Indirect effects on policy support					
Education	0.07	0.01	[0.05; 0.09]	8.435	<.001
Political orientation	−0.03	0.002	[-0.04; -0.03]	−17.394	<.001
Total effects on policy support					
Education	0.09	0.03	[0.01; 0.18]	2.847	.004
Political orientation	−0.06	0.01	[-0.07; -0.04]	−8.954	<.001

Note. N = 17,635; b = Unstandardized regression coefficient; *SE* = Standard error; *99% CI* = 99% Bootstrapped Confidence interval; z = z-score; p = p-value with a significance level of 5%.
[a]effect-coded variable with -1 = Lower education and 1 = Higher education. [b]grand-mean centered score with higher values indicating more right-leaning political orientation. [c]raw score with higher values indicating higher belief in climate change.

 Johannes Kotz, Helge Giese, Christian Breunig, Maj-Britt Sterba, Nathalie Brack, Patrick Dumont, Marija Taflaga, Javier Olivera, Lior Sheffer, Annika Werner, Anam Kuraishi, Wolfgang Gaissmaier

Supplementary Table S26

Table S26. Results of standardized path analysis on policy support to subsidize the purchase of electric vehicles with belief in climate change as mediator among citizens

Paths	β	*SE*	*99% CI*	*z*	*p*
Effects on belief in climate change					
Education[a]	0.15	0.02	[0.11; 0.19]	8.803	<.001
Political orientation[b]	−0.17	0.01	[-0.19; -0.15]	−21.426	<.001
Direct effects on policy support					
Education	0.01	0.02	[-0.03; 0.06]	0.766	.444
Political orientation	−0.03	0.01	[-0.05; -0.01]	−3.684	<.001
Belief in climate change[c]	0.25	0.01	[0.23; 0.27]	30.673	<.001
Indirect effects on policy support					
Education	0.04	0.004	[0.03; 0.05]	8.434	<.001
Political orientation	−0.04	0.002	[-0.05; -0.04]	−17.399	<.001
Total effects on policy support					
Education	0.05	0.02	[0.004; 0.10]	2.847	0.004
Political orientation	−0.07	0.01	[-0.09; -0.05]	−8.957	<.001

Note. N = 17,635; β = Standardized regression coefficient; *SE* = Standard error; *99% CI* = 99% Bootstrapped Confidence interval; z = z-score; p = p-value with a significance level of 5%.
[a]effect-coded variable with -1 = Lower education and 1 = Higher education. [b]z-standardized score with higher values indicating more right-leaning political orientation. [c]z-standardized score with higher values indicating higher belief in climate change.

Supplementary Table S27

Table S27. Results of unstandardized multigroup SEM on policy support to increase airplane ticket prices with belief in climate change as mediator

Paths	*b*	*SE*	*99% CI*	*z*	*p*
Politician Group					
Effects on belief in climate change					
Education[a]	0.27	0.12	[-0.04; 0.60]	2.203	.028
Political orientation[b]	−0.33	0.02	[-0.38; -0.27]	−15.776	<.001
Direct effects on policy support					
Education	0.17	0.14	[-0.19; 0.54]	1.198	.231
Political orientation	−0.10	0.03	[-0.17; -0.02]	−3.349	.001
Belief in climate change[c]	0.59	0.04	[0.47; 0.69]	13.572	<.001
Indirect effects on policy support					
Education	0.16	0.07	[-0.02; 0.36]	2.192	.028
Political orientation	−0.19	0.02	[-0.24; -0.14]	−10.285	<.001
Total effects on policy support					
Education	0.33	0.16	[-0.07; 0.73]	2.072	.038
Political orientation	−0.29	0.03	[-0.36; -0.22]	−10.784	<.001
Citizen Group					
Effects on belief in climate change					
Education[a]	0.23	0.03	[0.17; 0.30]	8.752	<.001
Political orientation[b]	−0.12	0.01	[-0.13; -0.10]	−22.017	<.001
Direct effects on policy support					
Education	−0.06	0.03	[-0.14; 0.03]	−1.764	.078
Political orientation	−0.07	0.01	[-0.09; -0.05]	−10.965	<.001
Belief in climate change[c]	0.27	0.01	[0.24; 0.29]	28.351	<.001
Indirect effects on policy support					
Education	0.06	0.01	[0.04; 0.08]	8.324	<.001
Political orientation	−0.03	0.002	[-0.04; -0.03]	−17.200	<.001
Total effects on policy support					
Education	0.004	0.03	[-0.08; 0.09]	0.123	.903
Political orientation	−0.10	0.01	[-0.12; -0.08]	−15.511	<.001
Contrasts of indirect effects between groups					
Education	0.10	0.07	[-0.08; 0.30]	1.328	.184
Political orientation	−0.16	0.02	[-0.21; -0.11]	−8.593	<.001

Note. N = 709 politicians and 17,696 citizens; b = Unstandardized regression coefficient; *SE* = Standard error; *99% CI* = 99% Bootstrapped Confidence interval; z = z-score; p = p-value with a significance level of 5%.
[a]effect-coded variable with -1 = Lower education and 1 = Higher education. [b]grand-mean centered score with higher values indicating more right-leaning political orientation. [c]raw score with higher values indicating higher belief in climate change.

Supplementary Table S28

Table S28. Results of standardized multigroup SEM on policy support to increase airplane ticket prices with belief in climate change as mediator

Paths	β	*SE*	*99% CI*	z	p
Politician Group					
Effects on belief in climate change					
Education[a]	0.17	0.08	[-0.03; 0.38]	2.203	.028
Political orientation[b]	−0.49	0.03	[-0.56; -0.41]	−15.776	<.001
Direct effects on policy support					
Education	0.09	0.07	[-0.10; 0.28]	1.198	.231
Political orientation	−0.12	0.04	[-0.22; -0.03]	−3.349	.001
Belief in climate change[c]	0.49	0.04	[0.40; 0.58]	13.572	<.001
Indirect effects on policy support					
Education	0.08	0.04	[-0.01; 0.19]	2.192	.028
Political orientation	−0.24	0.02	[-0.30; -0.18]	−10.285	<.001
Total effects on policy support					
Education	0.17	0.08	[-0.04; 0.38]	2.072	.038
Political orientation	−0.36	0.03	[-0.45; -0.27]	−10.784	<.001
Citizen Group					
Effects on belief in climate change					
Education[a]	0.15	0.02	[0.10; 0.19]	8.752	<.001
Political orientation[b]	−0.17	0.01	[-0.19; -0.15]	−22.017	<.001
Direct effects on policy support					
Education	−0.03	0.02	[-0.08; 0.01]	−1.764	.078
Political orientation	−0.09	0.01	[-0.11; -0.07]	−10.965	<.001
Belief in climate change[c]	0.22	0.01	[0.20; 0.24]	28.351	<.001
Indirect effects on policy support					
Education	0.03	0.004	[0.02; 0.04]	8.324	<.001
Political orientation	−0.04	0.002	[-0.04; -0.03]	−17.200	<.001
Total effects on policy support					
Education	0.002	0.02	[-0.04; 0.05]	0.123	.903
Political orientation	−0.13	0.01	[-0.15; -0.10]	−15.511	<.001
Contrasts of indirect effects between groups					
Education	0.05	0.04	[-0.04; 0.16]	1.328	.184
Political orientation	−0.20	0.02	[-0.26; -0.14]	−8.593	<.001

Note. $N = 709$ politicians and 17,696 citizens; β = Standardized regression coefficient; *SE* = Standard error; *99% CI* = 99% Bootstrapped Confidence interval; z = z-score; p = p-value with a significance level of 5%.
[a]effect-coded variable with -1 = Lower education and 1 = Higher education. [b]z-standardized score with higher values indicating more right-leaning political orientation. [c]z-standardized score with higher values indicating higher belief in climate change.

Supplementary Table S29

Table S29. Results of unstandardized multigroup SEM on policy support to subsidize the purchase of electric vehicles with belief in climate change as mediator

Paths	*b*	*SE*	*99% CI*	*z*	*p*
Politician Group					
Effects on belief in climate change					
Education[a]	0.27	0.12	[-0.04; 0.59]	2.240	.025
Political orientation[b]	−0.33	0.02	[-0.38; -0.27]	−16.201	<.001
Direct effects on policy support					
Education	−0.13	0.14	[-0.51; 0.24]	−0.881	.378
Political orientation	−0.07	0.03	[-0.15; 0.001]	−2.483	.013
Belief in climate change[c]	0.32	0.04	[0.21; 0.43]	7.539	<.001
Indirect effects on policy support					
Education	0.09	0.04	[-0.01; 0.20]	2.169	.030
Political orientation	−0.11	0.02	[-0.15; -0.07]	−6.451	<.001
Total effects on policy support					
Education	−0.04	0.15	[-0.44; 0.34]	−0.258	.796
Political orientation	−0.18	0.03	[-0.24; -0.11]	−7.142	<.001
Citizen Group					
Effects on belief in climate change					
Education[a]	0.24	0.03	[0.17; 0.30]	8.873	<.001
Political orientation[b]	−0.11	0.01	[-0.13; -0.10]	−21.388	<.001
Direct effects on policy support					
Education	0.02	0.03	[-0.06; 0.11]	0.759	.448
Political orientation	−0.02	0.01	[-0.04; -0.01]	−3.650	<.001
Belief in climate change[c]	0.29	0.01	[0.27; 0.32]	30.668	<.001
Indirect effects on policy support					
Education	0.07	0.01	[0.05; 0.09]	8.512	<.001
Political orientation	−0.03	0.002	[-0.04; -0.03]	−17.337	<.001
Total effects on policy support					
Education	0.09	0.03	[0.01; 0.18]	2.829	.005
Political orientation	−0.06	0.01	[-0.07; -0.04]	−8.894	<.001
Contrasts of indirect effects between groups					
Education	0.02	0.04	[-0.08; 0.14]	0.465	.642
Political orientation	−0.07	0.02	[-0.12; -0.03]	−4.376	<.001

Note. N = 713 politicians and 17,635 citizens; b = Unstandardized regression coefficient; *SE* = Standard error; *99% CI* = 99% Bootstrapped Confidence interval; z = z-score; p = p-value with a significance level of 5%.
[a]effect-coded variable with -1 = Lower education and 1 = Higher education. [b]grand-mean centered score with higher values indicating more right-leaning political orientation. [c]raw score with higher values indicating higher belief in climate change.

Supplementary Table S30

Table S30. Results of standardized multigroup SEM on policy support to subsidize the purchase of electric vehicles with belief in climate change as mediator

Paths	β	*SE*	*99% CI*	z	p
Politician Group					
Effects on belief in climate change					
Education[a]	0.17	0.08	[-0.02; 0.37]	2.240	.025
Political orientation[b]	−0.49	0.03	[-0.56; -0.41]	−16.201	<.001
Direct effects on policy support					
Education	−0.07	0.08	[-0.27; 0.13]	−0.881	.378
Political orientation	−0.09	0.04	[-0.19; 0.001]	−2.483	.013
Belief in climate change[c]	0.28	0.04	[0.18; 0.37]	7.539	<.001
Indirect effects on policy support					
Education	0.05	0.02	[-0.005; 0.11]	2.169	.030
Political orientation	−0.14	0.02	[-0.19; -0.08]	−6.451	<.001
Total effects on policy support					
Education	−0.02	0.08	[-0.23; 0.18]	−0.258	.796
Political orientation	−0.23	0.03	[-0.31; -0.15]	−7.142	<.001
Citizen Group					
Effects on belief in climate change					
Education[a]	0.15	0.02	[0.11; 0.19]	8.873	<.001
Political orientation[b]	−0.17	0.01	[-0.19; -0.15]	−21.388	<.001
Direct effects on policy support					
Education	0.01	0.02	[-0.03; 0.06]	0.759	.448
Political orientation	−0.03	0.01	[-0.05; -0.01]	−3.650	<.001
Belief in climate change[c]	0.25	0.01	[0.23; 0.27]	30.668	<.001
Indirect effects on policy support					
Education	0.04	0.004	[0.03; 0.05]	8.512	<.001
Political orientation	−0.04	0.002	[-0.05; -0.04]	−17.337	<.001
Total effects on policy support					
Education	0.05	0.02	[0.004; 0.10]	2.829	.005
Political orientation	−0.07	0.01	[-0.09; -0.05]	−8.894	<.001
Contrasts of indirect effects between groups					
Education	0.01	0.02	[-0.04; 0.07]	0.465	.642
Political orientation	−0.09	0.02	[-0.15; -0.04]	−4.376	<.001

Note. $N = 713$ politicians and 17,635 citizens; β = Standardized regression coefficient; *SE* = Standard error; *99% CI* = 99% Bootstrapped Confidence interval; z = z-score; p = p-value with a significance level of 5%.
[a]effect-coded variable with -1 = Lower education and 1 = Higher education. [b]z-standardized score with higher values indicating more right-leaning political orientation. [c]z-standardized score with higher values indicating higher belief in climate change.

Supplementary Table S31

Table S31. Results of standardized path analysis on policy support to increase airplane ticket prices with age and gender as predictors among citizens

Paths	β	*SE*	*99% CI*	z	p
Effects on belief in climate change					
Gender[a]	0.07	0.02	[0.03; 0.11]	4.603	<.001
Education[b]	0.13	0.02	[0.09; 0.18]	7.808	<.001
Age[c]	−0.05	0.01	[-0.07; -0.03]	−6.451	<.001
Political orientation[d]	−0.17	0.01	[-0.19; -0.15]	−21.404	<.001
Direct effects on policy support					
Gender	−0.07	0.02	[-0.11; -0.03]	−4.767	<.001
Education	−0.002	0.02	[-0.05; 0.04]	−0.102	.919
Age	0.10	0.01	[0.08; 0.12]	14.359	<.001
Political orientation	−0.09	0.01	[-0.11; -0.07]	−10.914	<.001
Belief in climate change[e]	0.23	0.01	[0.21; 0.25]	29.659	<.001
Indirect effects on policy support					
Gender	0.02	0.003	[0.01; 0.03]	4.522	<.001
Education	0.03	0.004	[0.02; 0.04]	7.560	<.001
Age	−0.01	0.002	[-0.02; -0.01]	−6.211	<.001
Political orientation	−0.04	0.002	[-0.05; -0.03]	−17.236	<.001
Total effects on policy support					
Gender	−0.05	0.02	[-0.09; -0.02]	−3.599	<.001
Education	0.03	0.02	[-0.02; 0.07]	1.629	.103
Age	0.09	0.01	[0.07; 0.11]	12.511	<.001
Political orientation	−0.13	0.01	[-0.15; -0.11]	−15.577	<.001

Note. N = 17,626; β = Standardized regression coefficient; *SE* = Standard error; *99% CI* = 99% Bootstrapped Confidence interval; z = z-score; p = p-value with a significance level of 5%.
[a]effect-coded variable with -1 = Male and 1 = Female. [b]effect-coded variable with -1 = Lower education and 1 = Higher education. [c]z-standardized score with higher values indicating older age. [d]z-standardized score with higher values indicating more right-leaning political orientation. [e]z-standardized score with higher values indicating higher belief in climate change.

Supplementary Table S32

Table S32. Results of standardized path analysis on policy support to subsidize the purchase of electric vehicles with age and gender as predictors among citizens

Paths	β	*SE*	*99% CI*	*z*	*p*
Effects on belief in climate change					
Gender[a]	0.07	0.02	[0.03; 0.11]	4.728	<.001
Education[b]	0.14	0.02	[0.09; 0.18]	7.897	<.001
Age[c]	−0.05	0.01	[-0.07; -0.03]	−6.489	<.001
Political orientation[d]	−0.17	0.01	[-0.19; -0.15]	−21.028	<.001
Direct effects on policy support					
Gender	−0.03	0.02	[-0.07; 0.004]	−2.335	.020
Education	−0.02	0.02	[-0.06; 0.03]	−0.942	.346
Age	−0.10	0.01	[-0.12; -0.08]	−13.626	<.001
Political orientation	−0.03	0.01	[-0.05; -0.01]	−4.347	<.001
Belief in climate change[e]	0.25	0.01	[0.22; 0.26]	30.690	<.001
Indirect effects on policy support					
Gender	0.02	0.004	[0.01; 0.03]	4.647	<.001
Education	0.03	0.004	[0.02; 0.04]	7.628	<.001
Age	−0.01	0.002	[-0.02; -0.01]	−6.272	<.001
Political orientation	−0.04	0.002	[-0.05; -0.04]	−17.196	<.001
Total effects on policy support					
Gender	−0.02	0.02	[-0.06; 0.02]	−1.129	.259
Education	0.02	0.02	[-0.03; 0.06]	0.965	0.334
Age	−0.11	0.01	[-0.13; -0.09]	−14.869	<.001
Political orientation	−0.07	0.01	[-0.10; -0.06]	−9.563	<.001

Note. $N = 17{,}562$; β = Standardized regression coefficient; *SE* = Standard error; *99% CI* = 99% Bootstrapped Confidence interval; z = z-score; p = p-value with a significance level of 5%.
[a]effect-coded variable with -1 = Male and 1 = Female. [b]effect-coded variable with -1 = Lower education and 1 = Higher education. [c]z-standardized score with higher values indicating older age. [d]z-standardized score with higher values indicating more right-leaning political orientation. [e]z-standardized score with higher values indicating higher belief in climate change.

Supplementary Table S33

Table S33. Ethics approval for surveys of politicians in the respective countries

Country	Ethics Committee	Reference Number	Date
Australia	Humanities and Social Sciences DERC of the ANU	$2022/408$	October 2022
Czechia	Commision for Ethics in Research of Faculty of Social Sciences at the Charles University	NA	March 2022
Denmark	Ethics Committee of the Department of Political Science of the University of Copenhagen	$2022-04$	February 2022
Flanders (Belgium)	Ethical Advice Committee of the Social and Human Sciences at the University of Antwerp	SHW_22_032	February 2022
Wallonia (Belgium)	Ethical Committee of the Social and Human Sciences at the Université libre de Bruxelles	$R2022/004$	March 2022
Germany	Ethical Advice Committee of the University of Konstanz	IRB statement $10/2021$	February 2021
Israel	University Committee for the Use of Human Subjects in Research at the Hebrew University of Jerusalem	29042022	April 2022
Luxembourg	LISER Research Ethics Committee	NA	August 2022
Netherlands	Ethics Committee of the Faculty of Social and Behavioral Sciences	$2022-PCJ-1477$	April 2022
Norway	Data Protection Services at the Norwegian Agency for Shared Services in Education and Research	770184	June 2022

Supplementary Table S34

Table S34. Full wordings of all items used in this study

Variable	Item wording	Code	Response format
Political orientation	"In politics, people sometimes talk of 'left' and 'right'. Where would you place yourself on this scale, where 0 means left and 10 means right?"	Q4	0 = Left<br>1–9 = intermediate<br>10 = Right
Policy preferences	"People hold different views on political issues. Please indicate to what extent you agree or disagree with the following political statements."<br>(a) To reduce carbon emissions, the price of airplane tickets should be increased<br>(b) The government should subsidize the purchase of electric vehicles	Q14_9<br>Q14_10	1 = Strongly disagree<br>2 = Disagree<br>3 = Somewhat disagree<br>4 = Neither agree nor disagree<br>5 = Somewhat agree<br>6 = Agree<br>7 = Strongly agree<br>88 = Don't know[a]
Belief in anthropogenic climate change	"To what extent do you agree or disagree with the following statements?"<br>Climate change is mostly due to human activity	Q80_1	1 = Strongly disagree<br>2 = Disagree<br>3 = Somewhat disagree<br>4 = Neither agree nor disagree<br>5 = Somewhat agree<br>6 = Agree<br>7 = Strongly agree
Gender	"What is your gender?"	Q16	1 = Male<br>2 = Female<br>3 = Other[b]
Age (citizens)	"What is your year of birth?"	Q17	Dropdown 1921–2010<br>(recoded to age in years)
Age (politicians)	Age category (pseudonymised)	Age_category	1 = Below median age<br>2 = Above median age<br>95 = Other[b]
Education	"We are trying to get a better understanding of both your own and your close contacts' social background. Would you be willing to provide us with the following information about yourself?"<br>Education level – self	Q52_1_1	1 = No or primary education<br>2 = Secondary education<br>3 = Higher non-university education<br>4 = University education<br>5 = Don't know[a]<br>6 = Prefer not to say[a]<br>7 = Not applicable[a]

Note. [a]Treated as missing value. [b]Pseudonymization category in politicians; treated as missing value.
