# Supplementary material for: Ideological polarization on anthropogenic climate change is stronger among politicians than among citizens across eight countries
Source: Commun Sustain. 2026 Jul 14;1(1):111. doi: 10.1038/s44458-026-00113-y (PMC13368580; doi:10.1038/s44458-026-00113-y)
Supplement: Supplementary file 1 — Transparent Peer Review file [file 44458_2026_113_MOESM1_ESM.pdf]

nature portfolio

Peer Review File

Ideological polarization on anthropogenic climate change is stronger among politicians than among citizens across eight countries

Corresponding Author: Mr Johannes Kotz

This file contains all editorial decision letters in order by version, followed by all author rebuttals in order by version.

Attachments originally included by the reviewers as part of their assessment can be found at the end of this file.

Version 0:

Decision Letter:

** Please ensure you delete the link to your author home page in this e-mail if you wish to forward it to your coauthors **

Dear Mr Kotz,

Your manuscript titled "Political Polarization of Climate Belief Is Stronger Among Politicians Than Among Citizens Across Eight Countries" has now been seen by 3 reviewers, and we include their comments at the end of this message. They find your work of interest, but some important points are raised. We are interested in the possibility of publishing your study in Communications Sustainability, but would like to consider your responses to these concerns and assess a revised manuscript before we make a final decision on publication.

We therefore invite you to revise and resubmit your manuscript, along with a point-by-point response that takes into account the points raised. Please highlight all changes in the manuscript text file.

In particular, for publication in Communications Sustainability, we request that you (1) deepen theoretical integration, (2) clarify all analytical assumptions, and (3) expand on implications for climate communications and policy.

Please submit your point-by-point responses as a separate file, distinct from your cover letter where you can add responses to the Editors' comments that you do not want to be made available to the reviewers. Word files are preferred. We recommend that any figures, tables or graphs that are included in the response to reviewers are also included in the main article or Supplementary Information.

We are committed to providing a fair and constructive peer-review process. Please don't hesitate to contact us if you wish to discuss the revision in more detail.

Please use the following link to submit your revised manuscript, point-by-point response to the reviewers' comments (which should be in a separate document to any cover letter), a tracked-changes version of the manuscript (as a PDF file) and the completed checklist:
Link Redacted
** This url links to your confidential home page and associated information about manuscripts you may have submitted or be reviewing for us. If you wish to forward this email to co-authors, please delete the link to your homepage first **

We hope to receive your revised paper within six weeks; please let us know if you aren't able to submit it within this time so that we can discuss how best to proceed. If we don't hear from you, and the revision process takes significantly longer, we may close your file. In this event, we will still be happy to reconsider your paper at a later date, as long as nothing similar has been accepted for publication at Communications Sustainability or published elsewhere in the meantime.

Please do not hesitate to contact us if you have any questions or would like to discuss these revisions further. We look forward to seeing the revised manuscript and thank you for the opportunity to review your work.

Best regards,

Katya Rhodes, PhD
Editorial Board Member
Communications Sustainability

Martina Grecequet, PhD
Consulting Editor,
Communications Sustainability
Senior Editor,
Communications Earth & Environment

EDITORIAL POLICIES AND FORMATTING

We ask that you ensure your manuscript complies with our editorial policies. Please ensure that the following formatting requirements are met, and any checklist relevant to your research is completed and uploaded as a Related Manuscript file type with the revised article.

For Manuscripts that fall into the following fields:
• Behavioural and social science
• Ecological, evolutionary & environmental sciences
• Life sciences
An updated and completed version of our Reporting Summary must be uploaded with the revised manuscript
You can download the form here:
https://www.nature.com/documents/nr-reporting-summary.pdf

Furthermore, please align your manuscript with our format requirements, which are summarized on the following checklist: Communications Sustainability formatting checklist

and also in our style and formatting guide Communications Sustainability formatting guide .

*** DATA: Communications Sustainability endorses the principles of the Enabling FAIR data project (http://www.copdess.org/enabling-fair-data-project/). We ask authors to make the data that support their conclusions available in permanent, publicly accessible data repositories. (Please contact the editor if you are unable to make your data available).

All Communications Sustainability manuscripts must include a section titled "Data Availability" at the end of the Methods section or main text (if no Methods). More information on this policy, is available at http://www.nature.com/authors/policies/data/data-availability-statements-data-citations.pdf.

In particular, the Data availability statement should include:
- Unique identifiers (such as DOIs and hyperlinks for datasets in public repositories)
- Accession codes where appropriate
- If applicable, a statement regarding data available with restrictions
- If a dataset has a Digital Object Identifier (DOI) as its unique identifier, we strongly encourage including this in the Reference list and citing the dataset in the Data Availability Statement.

DATA SOURCES: All new data associated with the paper should be placed in a persistent repository where they can be freely and enduringly accessed. We recommend submitting the data to discipline-specific, community-recognized repositories, where possible and a list of recommended repositories is provided at http://www.nature.com/sdata/policies/repositories.

If a community resource is unavailable, data can be submitted to generalist repositories such as figshare or Dryad Digital Repository. Please provide a unique identifier for the data (for example a DOI or a permanent URL) in the data availability statement, if possible. If the repository does not provide identifiers, we encourage authors to supply the search terms that will return the data. For data that have been obtained from publicly available sources, please provide a URL and the specific data product name in the data availability statement. Data with a DOI should be further cited in the methods reference section.

Please refer to our data policies at http://www.nature.com/authors/policies/availability.html.

REVIEWER COMMENTS:

Reviewer #1 (Remarks to the Author):

This aim of this paper is to compare systematically and cross-nationally, polarization in climate belief among politicians and citizens. The empirical scope of the study is eight developed democratic countries with harmonized survey datasets, and the application of a range of competently executed statistical techniques, including regression and path (mediation) analyses. The paper's findings aligns with an extensive literature that establishes that right leaning politicians and citizens show weaker belief in human-driven climate change. The novelty it adds to this literature is the finding that for politicians across the 8 countries in the study, belief in anthropogenic climate change is more polarized, than among citizens.

That said, the comparison between citizens and politicians is framed largely in terms of individual-level beliefs and attitudes, which overlooks important political–economic and institutional considerations that likely also shape how politicians express policy support and belief in climate change. Relatedly, while the paper draws on a broad literature on climate belief polarization, it lacks a focused review of relevant and/or conflicting studies on the relationship between polarization, climate beliefs, and climate policy support. Given the topic, the paper would also benefit from complimenting behavioral science and psychology references with a review of literature related to the political economy of climate change in reference to elite actors, ideology., and climate skepticism. Strengthening this conceptual background would help situate the empirical findings more clearly within existing debates and sharpen the interpretation of the citizen–politician comparison. My major comments are geared mostly toward strengthening the interpretation of results and better situating the study in current literature, with a few minor comments that I made note of in my reading.

MAJOR COMMENTS
1. A central contribution of the paper is the finding that climate belief polarization is stronger among politicians than among citizens. The analysis risks implying that right-leaning politicians believe less in anthropogenic climate change in a purely factual or epistemic sense, rather than that they may express climate beliefs strategically in response to political–economic incentives (for example party positions, lobbying, campaign finance, or fossil fuel interests). This framing treats politicians as individuals suffering from cognitive errors (biases) rather than rational actors who might know the science is real but answer the question strategically. I recommend that the authors engage with political economy and elite behavior literatures and integrate these perspectives into the discussion of their findings to clarify why expressed climate beliefs may diverge more sharply among politicians than among citizens. This may also impact the author's proposed solutions.

2. Related to the previous point, in the path analysis (Section 2.3), it is assumed that belief in climate change explains how ideology translates into policy support. These paths are acknowledged as theoretical, however, particularly for politicians, it may be important to consider how statements of climate belief may instead function as discursive justifications for pre-existing party or policy positions, rather than as drivers of policy preferences. This interpretation raises questions about the causal ordering implied by the path model, especially given that the main contribution of the paper is the comparison between citizens and politicians. Better rooting of these theoretical relationships in established literature would strengthen the paper and may uncover theoretical paths that differ between citizens and politicians.

3. The study operationalizes polarization as the strength of the relationship between political orientation (left–right) and belief in anthropogenic climate change - the steeper the slope = more polarization. This approach may miss a more nuanced interpretation of polarization in these contexts. For example, there are studies showing variation between the sides of the political spectrum (ex. Berkebile-Weinberg et al. 2024 which is cited in the discussion but not the literature review - it is a very relevant and similar study also published recently in Nature Communications). As polarization in climate belief is already well established in the literature, the authors could strengthen thier contribution by exploring their data set in ways that can distill the nuances of climate belief along ideological lines.
Citation: M. Berkebile-Weinberg, D. Goldwert, K. C. Doell, J. J. Van Bavel, M. Vlasceanu, The differential impact of climate interventions along the political divide in 60 countries. Nat Commun 15, 3885 (2024).

MINOR COMMENTS
a. Sentence in line 89-95 is unclear
b. In Table , it would be interesting to see how the sample compares to available census or administrative data on the population of elected officials in each country.
c. The paper concludes with saying "we show that belief in anthropogenic climate change is heavily polarized..." but I am curious as to how the study defines heavy vs. light polarization.
d. The study finds that education plays a minimal role, but is also tested using a binary education measure which may reduce sensitivity and underestimate its role.

Reviewer #2 (Remarks to the Author):

This study makes use of an impressive sample of citizens and politicians across eight countries to examine the political polarization of climate change beliefs. They find that politicians are much more polarized than are citizens, meaning politicians – especially those who are on the political right – do not reflect the beliefs of those they represent. I enjoyed reading this paper and was particularly impressed by the interesting results and the large multi-country sample consisting of citizens and politicians.

Major suggestions/comments on theory and background:
In your analysis, you claim that the discordance between citizens and elites on climate beliefs is driven primarily by more

right-wingers – right-wing politicians do not believe in climate change as much as their right-wing constituents. However, in Figure 1a, there also appears to be a stark difference between left-wing politicians and left-wing citizens. Can you say more about why you think this is driven more by right-wingers? Do you have any analyses to back this up?

You have done a very convincing job in establishing that political elites are much more polarized than are citizens. In the discussion, it would be wonderful if the researchers spent more time explaining why they think this is the case. According to many researchers, citizens merely adopt the political views of elites and do not form political attitudes and beliefs on their own (unless they are politically sophisticated; e.g., Van Boven & Sherman, 2021; Sniderman & Bullock, 2004). The data here show that this might not always be the case, regardless of education/political sophistication. Why? Are citizens capable of forming political beliefs without elite input? Is climate change a special issue in which elites influence public opinion to a lesser degree than other political issues? Or does it trickle down in other ways (e.g., specific beliefs may differ, but hostility – or affective polarization – among politicians and citizens mirror one another)? It would be great if the authors could touch more on these issues and also incorporate more literature regarding how citizens form their political opinions (e.g., bottom up vs top down).

Results
In the main text (possibly in the figure for each country), please add the n for each country. Additionally, were any countries more represented than others? If so, is there a possibility that the results of the primary analyses reported and plotted in Figure 1a are more influenced by one country compared to the others? How are the authors dealing with this issue statistically? One potential solution could be to use a multi-level model.

The way in which unstandardized and standardized betas are reported is slightly confusing – why do they have different p-values? If the p-values do not match, it means the coding of one or more variables is slightly off in the standardized analysis. Once that is fixed, the authors can provide one p-value that corresponds to both the unstandardized and standardized coefficients, which will read much smoother.

When describing the mediation, starting with the b path is confusing (e.g., the link between climate belief and policy support). Why not start with the total effect or the a path?

Please refrain from comparing the size of coefficients unless you are doing a direct test (e.g., page 9, where the authors claim “belief in anthropogenic climate change showed the strongest direct effect”). Please also be careful about using the word “direct effect” when talking about any path that is not the c’.

The authors should be careful about claiming that an effect with a p-value of <.001 is negligible (e.g., page 9). There is no need to downplay the (very) significant indirect effects among citizens, given that the multi-group SEM indicates that the indirect effects are stronger among politicians.

Minor comments:
Since the citizen and politicians samples were initially discussed independently, I assumed that separate analyses would be reported. Please state more clearly at the beginning of the results section that the primary analyses are conducted on a sample that includes both citizens and politicians.

It would be great if the authors added more information about the sample in the main text (as opposed to the method), particularly the impressive politician sample. For example, how they were recruited, and what level of government they are in.

Please state more clearly when you are reporting a two or three-way interaction. For example, at the top of page 8, the authors say, “Although the difference in polarization between politicians and citizens varied across countries.” It would be much clearer if they also described this as a three-way interaction between ideology, politician vs. citizen status, and country.

On page 6, the authors report that there is a significant interaction between political polarization and politician vs. citizen status. In the following sentence (“While right-leaning citizens show slightly lower belief than left-leaning citizens, belief in climate change was significantly lower”), it would be helpful if they could additionally report the simple effects of ideology on belief in climate change for both citizens and politicians (e.g., what are the slopes of the blue and red lines in Figure 1a). Is the following sentence (“Accordingly, the paired comparison of the political orientation effect resulted in a significant difference between the groups ($t(18726) = -7.118$, $p < .001$), with $b = -0.35$ and $\beta = -0.53$ (both $p < .001$) for politicians and $b = -0.12$ as well as $\beta = -0.18$ (both $p < .001$) for citizens”) describing the simple effects? If so, can the authors state more clearly that these are simple effects meant to decompose the significant interaction?

In the mediation figures, the a and the b paths specify the β, while the total effect only reports the standardized coefficient in parentheses.

Could the authors present the order of supplementary tables in the same order they are referenced in the main text (e.g., Table S10 is currently mentioned before Table S9)?

It is unclear to me why political extremity was controlled for, and how it was calculated. Was political extremity calculated using the political ideology measure, and if so, is it appropriate to use it in a model that also includes political ideology? It seems to be that the model would then contain redundant variables. A more in depth explanation would help clear this up.

If the authors can address these comments, I believe that the manuscript should be accepted.

Reviewer #3 (Remarks to the Author):

Thank you for the opportunity to review this well-written and impressive manuscript. It investigates the impact of political orientation on belief in human-driven climate change and support for mitigation policies among citizens and politicians in eight developed democracies. It compares the level of polarization of belief in anthropogenic climate change and policy support among the public and politicians, and it evaluates the impact of higher education levels among both groups as a proxy for science literacy. It nicely reviews the existing related literature to motivate its central research questions. The manuscript makes a compelling point that few studies have explored the level of polarization among politicians compared to their constituencies on this topic, nor the relative impact of political orientation in driving belief polarization across a large sample of countries and politicians. The large cross-national dataset employed for the analyses is impressive, and the consistent finding that polarization is higher among politicians than the public is novel and important. The findings also demonstrate the powerful impact of political ideology on belief human-caused climate change for both groups, which is the strongest predictor of support for mitigation policies. The impact of political orientation on these beliefs is not conditional on education. Additionally, the analyses suggest that belief in human-driven climate change mediates the relationship between political orientation and support for mitigation policies for citizens and politicians. The results and analyses are clearly presented in the text and figures. The discussion section clearly addresses the implications of the findings for generating greater consensus for policy action among skeptical audiences. However, are there other ways in addition to emphasizing the scientific consensus on the topic to increase belief in human caused climate change? Is it worth mentioning the challenges of building consensus on polarized and politicized issues that can generate motivated reasoning and activate partisan group norms when people are processing scientific messages, and/or ways to break through this persistent ideological/partisan polarization? Overall, this manuscript makes a significant contribution to the existing literature on political polarization on climate change, and I look forward to its publication.

** Visit Nature Portfolio's author and reviewers' website at www.nature.com/authors for information about policies, services and author benefits**

Communications Sustainability is committed to improving transparency in authorship. As part of our efforts in this direction, we are now requesting that all authors identified as ‘corresponding author’ create and link their Open Researcher and Contributor Identifier (ORCID) with their account on the Manuscript Tracking System prior to acceptance. ORCID helps the scientific community achieve unambiguous attribution of all scholarly contributions. You can create and link your ORCID from the home page of the Manuscript Tracking System by clicking on ‘Modify my Springer Nature account’ and following the instructions in the link below. Please also inform all co-authors that they can add their ORCIDs to their accounts and that they must do so prior to acceptance.
https://www.springernature.com/gp/researchers/orcid/orcid-for-nature-research

For more information please visit http://www.springernature.com/orcid

If you experience problems in linking your ORCID, please contact the Platform Support Helpdesk.

Version 1:

Decision Letter:

** Please ensure you delete the link to your author home page in this e-mail if you wish to forward it to your coauthors **

Dear Mr Kotz,

Your manuscript titled "Political Polarization of Climate Belief Is Stronger Among Politicians Than Among Citizens Across Eight Countries" has now been seen by our reviewers, whose comments appear below. In light of their advice we are delighted to say that we are happy, in principle, to publish a suitably revised version in Communications Sustainability.

We therefore invite you to revise your paper one last time to address the remaining concerns of our reviewers. In particular, we request that you avoid causal-sounding language (such as "effect", "influence", or "impact") throughout the manuscript when discussing results unless adequately supported, and appropriately caveated causal claims.

At the same time we ask that you edit your manuscript to comply with our format requirements and to maximise the accessibility and therefore the impact of your work.

EDITORIAL REQUESTS:

Please review our specific editorial comments and requests regarding your manuscript in the attached "Editorial Requests Table".

*****Please take care to match our formatting and policy requirements. We will check revised manuscript and return manuscripts that do not comply. Such requests will lead to delays. *****

Please outline your response to each request in the right hand column. Please upload the completed table with your manuscript files as a Related Manuscript file.

If you have any questions or concerns about any of our requests, please do not hesitate to contact me.

SUBMISSION INFORMATION:

In order to accept your paper, we require the files listed at the end of the Editorial Requests Table; the list of required files is also available at https://www.nature.com/documents/commsj-file-checklist.pdf .

OPEN ACCESS:

Communications Sustainability is a fully open access journal. Articles are made freely accessible on publication. For further information about article processing charges, open access funding, and advice and support from Nature Portfolio, please visit https://www.nature.com/commssustain/open-access

At acceptance, you will be provided with instructions for completing the open access licence agreement on behalf of all authors. This grants us the necessary permissions to publish your paper. Additionally, you will be asked to declare that all required third party permissions have been obtained, and to provide billing information in order to pay the article-processing charge (APC).

Please use the following link to submit the above items:
Link Redacted
** This url links to your confidential home page and associated information about manuscripts you may have submitted or be reviewing for us. If you wish to forward this email to co-authors, please delete the link to your homepage first **

We hope to hear from you within two weeks; please let us know if you need more time.

Best regards,

Katya Rhodes, PhD
Editorial Board Member
Communications Sustainability

Martina Grecequet, PhD
Consulting Editor,
Communications Sustainability
Senior Editor,
Communications Earth & Environment

REVIEWERS' COMMENTS:

Reviewer #1 (Remarks to the Author):

I thank the authors for their thoughtful revisions. The revised manuscript is significantly strengthened, particularly in its engagement with political economy and elite behavior perspectives, additional analyses examining divergence across ideological groups, and the clearer acknowledgment of the limitations surrounding causal interpretation in the mediation analyses. Overall, I believe the authors have addressed my major concerns satisfactorily.
There are a couple areas that could possibly use some further attention or clarification if the authors are interested, but I would not say a full revision is necessary.
1. The revised results section adds a nuanced interpretation of the asymmetries across the ideological spectrum but still operationalizes polarization as linear slope - so comments such as in line 409: "The gaps between politicians and their electorate widen towards the ideological extremes" is more of a geometric property of two linear relationships with different slopes, rather than evidence of distinct nonlinear patterns of polarization. Perhaps future work by the authors could look more closely at non-linear relationships.
2. I still view some aspects of the mediation interpretation as theoretically constrained by the design, particularly given the possibility that policy positioning may shape expressed climate beliefs among politicians rather than the reverse. However, the revised manuscript now explicitly acknowledges this limitation and frames the mediation analyses more cautiously.
Thank you for the opportunity to review the revised version of this manuscript.

Reviewer #2 (Remarks to the Author):

The paper has been much improved. I only have a few small comments:

The title for the section “Stronger polarization of belief in anthropogenic climate change among politicians” would be clearer if it said “Stronger polarization of belief in anthropogenic climate change among politicians compared to citizens” to make the comparison more salient in the section title.

Sometimes, between the b and the standardized b, the authors put the word “and”, which is not needed. Just list the coefficients.

On page 9, when the authors say “However, the direction of the interaction effect was consistent across countries (see Supplementary Tables S11 and S12 for standardized slopes and contrasts between groups for each country),” they should further clarify this by also stating something along the lines of “in that slopes among politicians were steeper than those among citizens in all countries”.

I believe that this paper should be accepted, given that these small changes are made.

Reviewer #3 (Remarks to the Author):

The authors have clearly responded to the suggestions and requests from all reviewers. I was enthusiastic about the original version of this manuscript, and the thoughtful suggestions and responses to the other reviewers' comments have resulted in an even stronger piece of research. I recommend it be published.

** Visit Nature Portfolio's author and reviewers' website at www.nature.com/authors for information about policies, services and author benefits**

Open Access This Peer Review File is licensed under a Creative Commons Attribution 4.0 International License, which permits use, sharing, adaptation, distribution and reproduction in any medium or format, as long as you give appropriate credit to the original author(s) and the source, provide a link to the Creative Commons license, and indicate if changes were made.
In cases where reviewers are anonymous, credit should be given to 'Anonymous Referee' and the source.
The images or other third party material in this Peer Review File are included in the article’s Creative Commons license,

unless indicated otherwise in a credit line to the material. If material is not included in the article's Creative Commons license and your intended use is not permitted by statutory regulation or exceeds the permitted use, you will need to obtain permission directly from the copyright holder.
To view a copy of this license, visit https://creativecommons.org/licenses/by/4.0/

REVIEWER COMMENTS:

Reviewer #1 (Remarks to the Author):

This aim of this paper is to compare systematically and cross-nationally, polarization in climate belief among politicians and citizens. The empirical scope of the study is eight developed democratic countries with harmonized survey datasets, and the application of a range of competently executed statistical techniques, including regression and path (mediation) analyses. The paper's findings aligns with an extensive literature that establishes that right-leaning politicians and citizens show weaker belief in human-driven climate change. The novelty it adds to this literature is the finding that for politicians across the 8 countries in the study, belief in anthropogenic climate change is more polarized, than among citizens.

That said, the comparison between citizens and politicians is framed largely in terms of individual-level beliefs and attitudes, which overlooks important political–economic and institutional considerations that likely also shape how politicians express policy support and belief in climate change. Relatedly, while the paper draws on a broad literature on climate belief polarization, it lacks a focused review of relevant and/or conflicting studies on the relationship between polarization, climate beliefs, and climate policy support. Given the topic, the paper would also benefit from complimenting behavioral science and psychology references with a review of literature related to the political economy of climate change in reference to elite actors, ideology., and climate skepticism. Strengthening this conceptual background would help situate the empirical findings more clearly within existing debates and sharpen the interpretation of the citizen–politician comparison. My major comments are geared mostly toward strengthening the interpretation of results and better situating the study in current literature, with a few minor comments that I made note of in my reading.

MAJOR COMMENTS

1. A central contribution of the paper is the finding that climate belief polarization is stronger among politicians than among citizens. The analysis risks implying that right-leaning politicians believe less in anthropogenic climate change in a purely factual or epistemic sense, rather than that they may express climate beliefs strategically in response to political–economic incentives (for example party positions, lobbying, campaign finance, or fossil fuel interests). This framing treats politicians as individuals suffering from cognitive errors (biases) rather than rational actors who might know the science is real but answer the question strategically. I recommend that the authors engage with political economy and elite behavior literatures and integrate these perspectives into the discussion of their findings to clarify why expressed climate beliefs may diverge more sharply among politicians than among citizens. This may also impact the author's proposed solutions.

Answer: We thank the reviewer for this insightful observation. We agree that expressed climate beliefs among politicians may partly reflect strategic position-taking in response to political and economic incentives, rather than purely epistemic disagreement about the scientific evidence. In response, we revised the discussion section to more explicitly engage with political economy and elite behavior perspectives. The revised text clarifies that politicians face institutional incentive structures, such as party discipline, electoral competition, and lobbying pressures, that citizens typically do not encounter when expressing their views. These incentives can encourage politicians to adopt more polarized public positions regardless of their private assessment of climate science.

By highlighting these mechanisms, we aim to avoid interpreting elite polarization solely as a reflection of individual cognitive biases and instead situate it within the broader literature on strategic elite behavior and political incentives. This framing also informs the discussion of potential solutions by emphasizing that reducing elite polarization may require institutional or structural interventions, in addition to informational approaches.

The revised manuscript now states (lines 640-682):

> “However, beyond misperception of public opinion [34,35] or stronger ideological influences on issue attitudes [36], the stronger polarization of climate beliefs among politicians could also be explained by strategic position-taking rather than actual epistemic beliefs about climate change. Politicians face incentive structures such as party constraints, electoral incentives or lobbying pressures [37, 38] that citizens do not have when stating opinions, potentially leading to more extreme expressions of climate opinions regardless of their personal assessment of the science. Such polarized elite positions can shape voters’ attitudes about climate change [11, 39] and drive ideological and affective polarization among the public as well, particularly among politically interested citizens [40, 41]. Our findings that politicians are more polarized than citizens are consistent with models of top-down opinion formation [15] predicting that elite cues influence citizen opinion, not that citizens will mirror elite positions in full. Citizens form political attitudes through multiple channels beyond elite cues, including socialization, issue salience, personal experience, other sources of information and self interest, which attenuates the degree to which elite-level polarization is transmitted to the public [42-44]. Still, increasingly partisan elite positions may have driven public skepticism towards climate science as well [12]. Addressing the political incentives and ideological constraints that are potentially driving elite polarization requires structural solutions alongside epistemic ones, such as reducing financial and lobbying incentives for climate skepticism, or reforming electoral systems to reduce re-election pressures that

> reward partisan positioning. Informing right-leaning citizens that their representatives' climate positions diverge from their own could further encourage them to pressure politicians or vote accordingly, increasing congruence between politicians' positions and voters' preferences."

We believe this revision clarifies that our interpretation of elite polarization incorporates both strategic incentives and mechanisms of elite influence on public opinion.

2. Related to the previous point, in the path analysis (Section 2.3), it is assumed that belief in climate change explains how ideology translates into policy support. These paths are acknowledged as theoretical, however, particularly for politicians, it may be important to consider how statements of climate belief may instead function as discursive justifications for pre-existing party or policy positions, rather than as drivers of policy preferences. This interpretation raises questions about the causal ordering implied by the path model, especially given that the main contribution of the paper is the comparison between citizens and politicians. Better rooting of these theoretical relationships in established literature would strengthen the paper and may uncover theoretical paths that differ between citizens and politicians.

Answer: We thank the reviewer for this thoughtful point. We agree that the causal ordering in the path model is particularly uncertain for politicians, and that expressed climate beliefs may function as discursive justifications for party positions rather than as epistemic drivers of policy preferences. In addition to the discussion paragraph mentioned above introducing political incentives as possible drivers for belief expression about climate change, we also address in the limitations section that the causal ordering between climate belief and policy support may be reversed for politicians relative to citizens. We note that the citizen-based theoretical framework of climate beliefs shaping policy support is well established in the literature and is used in our study to keep mediation analyses comparable between citizens and politicians, while acknowledging that the underlying causal structure may differ between politicians and citizens. Since our observational data cannot distinguish between these causal orderings, we flag this as an important direction for future research using experimental or longitudinal designs with elite samples.

The revised manuscript now states (lines 762-787):

> "Finally, this study uses observational data. Therefore, causal pathways in mediation analyses are theoretical assumptions, justified by a broader influence of political orientation on support for mitigation policies compared to a more direct impact of the closely related belief in climate change. This causal ordering of both climate change perceptions and political orientation shaping support for climate action is an established

theoretical approach in the literature on citizens [6, 56–58]. However, among politicians, if expressed climate beliefs reflect strategic positioning in line with party stances on climate policy rather than genuine epistemic states, then policy preferences may drive expressed climate beliefs rather than the reverse. Since our observational data cannot distinguish between these two causal orderings, we applied the same theoretical framework to both politicians and citizens to keep the mediation analyses comparable. Future research using experimental or longitudinal designs could help establish the direction of this relationship among political elites."

We believe these additions directly address the reviewer's concern about the causal assumptions implied by the path model and the theoretical differences that may exist between politicians and citizens.

3. The study operationalizes polarization as the strength of the relationship between political orientation (left–right) and belief in anthropogenic climate change - the steeper the slope = more polarization. This approach may miss a more nuanced interpretation of polarization in these contexts. For example, there are studies showing variation between the sides of the political spectrum (ex. Berkebile-Weinberg et al. 2024 which is cited in the discussion but not the literature review - it is a very relevant and similar study also published recently in Nature Communications). As polarization in climate belief is already well established in the literature, the authors could strengthen thier contribution by exploring their data set in ways that can distill the nuances of climate belief along ideological lines.
Citation: M. Berkebile-Weinberg, D. Goldwert, K. C. Doell, J. J. Van Bavel, M. Vlasceanu, The differential impact of climate interventions along the political divide in 60 countries. Nat Commun 15, 3885 (2024).

Answer: We thank the reviewer for this helpful suggestion and agree that examining variation across the political spectrum provides important nuance beyond the overall slope. We have incorporated the Berkebile-Weinberg et al. (2024) study into the literature review, noting its relevance to the present work. To address the concern about asymmetry, we conducted additional analyses examining marginal means and contrasts at ±1 SD of political orientation separately for politicians and citizens and reported them in the results section (lines 308-391). We find that the gap in climate belief between left and right is larger among politicians than among citizens. Furthermore, we also find that left politicians have stronger beliefs than left citizens, even though both are on the higher end of the scale. Among right politicians, we find lower climate beliefs than their electorate. We also note that the gap between politicians and citizens widens towards the extremes. We furthermore added the procedure of estimating marginal means and contrast analyses to the methods section (lines 956-963). We also explicitly mention in the discussion section where

left and right politicians are on the belief scale and address the gap between politicians and citizens (lines 594-603).

The revised manuscript now states in the result section (lines 308-391):

> “However, the two-way interaction between political orientation and politician/citizen status indicates that political polarization of belief in anthropogenic climate change was stronger among politicians than citizens (b = -0.12, β = -0.18, p < .001), as illustrated in Fig. 1. Simple slope analyses confirmed a stronger association of political orientation with belief in climate change among politicians (b = -0.35 and β = -0.53, p < .001) than among citizens (b = -0.12 and β = -0.18, p < .001) and the slopes differed significantly between those groups (t(18,932) = -7.118, p < .001). Estimated marginal means and contrasts at ±1 standard deviation from the sample mean of political orientation (2.86 and 7.6 on a scale from ’0’ to ’10’; see Supplementary Table S9) further illustrate the divergence in the political orientation slope between politicians and citizens. While right-leaning citizens reported only slightly lower belief than left-leaning citizens ($\Delta_{\text{left-right}}$ = 0.56, t(18,932) = 17.307, p < .001), politicians exhibited a much steeper ideological divide, with substantially lower belief among right-leaning politicians and very strong belief among left-leaning politicians ($\Delta_{\text{left-right}}$ = 1.32, t(18,932) = 17.949, p < .001). This asymmetry was driven by divergence at both ends of the spectrum. On the left, politicians expressed stronger climate beliefs than citizens ($\Delta_{\text{politician-citizen}}$ = 0.752, t(18,932) = 10.157, p < .001), though both were on the higher end of the scale ($M_{\text{Politicians,left}}$ = 6.21; $M_{\text{Citizens,left}}$ = 5.46). On the right, the pattern reversed: Politicians expressed weaker belief in climate change than citizens ($\Delta_{\text{politician-citizen}}$ = -0.362, t(18,932) = -2.460, p = .014), with both groups above but closer to the scale midpoint ($M_{\text{Politicians,right}}$ = 4.53; $M_{\text{Citizens,right}}$ = 4.89), suggesting lower acceptance of human-driven climate change. As Fig. 1 illustrates, the gaps between politicians and their electorate widened towards the ideological extremes.“

MINOR COMMENTS

a. Sentence in line 89-95 is unclear

Answer: We thank the reviewer for pointing out this unclear sentence. We have split the sentence in two and hopefully made it clearer (lines 088-095):

> “Belief in anthropogenic climate change has become politically polarized, that is, opposing groups have adopted increasingly divergent stances on the issue [7]. This polarization is evident among right-leaning individuals,

who show significantly lower belief in climate change [6, 8, 9] and lower trust in environmental scientists [10] than left-leaning individuals."

b. In Table , it would be interesting to see how the sample compares to available census or administrative data on the population of elected officials in each country.

Answer: We thank the reviewer for pointing out the need to give more detail about the politician sample. We now report representativeness of our politician sample compared to their parliaments regarding gender, age, seniority, and party ideology. We also added Supplementary Tables S3 and S4 to give detailed information.

The revised manuscript now states in the results section (lines 195-215):

> "All country teams of this project (except for Germany) made efforts to reach out to all national members of parliament via e-mail. In Germany, given the large parliament size, a sampled population of members of parliament at the national level was targeted, ensuring representativeness of parliament in terms of gender, party, and incumbent status. In addition, Belgium, a federalized country, included regional politicians in their target populations from Flanders and Wallonia. Supplementary Tables S3 and S4 display the representativeness of the full politician sample compared to their respective countries' parliaments regarding gender, age, seniority, and party ideology. Gender and age distributions in our sample are similar to the target population with few exceptions (e.g. higher percentage of females in Israel). Seniority is overall slightly lower than in the respective parliaments, and we have a slightly lower participation rate among the right-wing in some countries."

c. The paper concludes with saying "we show that belief in anthropogenic climate change is heavily polarized..." but I am curious as to how the study defines heavy vs. light polarization.

Answer: We thank the reviewer for pointing out this vague formulation. We left out "heavily" and specified only that climate change is "politically" polarized (lines 788-792).

d. The study finds that education plays a minimal role, but is also tested using a binary education measure which may reduce sensitivity and underestimate its role.

Answer: We thank the reviewer for pointing out this methodological issue. We used a binary measure because treating education as an interval-scaled measure would not be statistically justifiable since the intervals cannot be assumed to be identical. Treating education as a categorical factor with 4 levels instead would result in the loss of information about the rank order of the education levels. Furthermore, among politicians, the lowest education levels are scarcely populated, so we would introduce even more uncertainty if we analyzed each level separately. Since it is

common in political science literature to compare university-level educated participants with lower educated ones, we decided to do this in our study as well. Nonetheless, we acknowledge the limitations of the binary education measure in lines 749-756.

Reviewer #2 (Remarks to the Author):

This study makes use of an impressive sample of citizens and politicians across eight countries to examine the political polarization of climate change beliefs. They find that politicians are much more polarized than are citizens, meaning politicians – especially those who are on the political right – do not reflect the beliefs of those they represent. I enjoyed reading this paper and was particularly impressed by the interesting results and the large multi-country sample consisting of citizens and politicians.

Major suggestions/comments on theory and background:

1. In your analysis, you claim that the discordance between citizens and elites on climate beliefs is driven primarily by more right-wingers – right-wing politicians do not believe in climate change as much as their right-wing constituents. However, in Figure 1a, there also appears to be a stark difference between left-wing politicians and left-wing citizens. Can you say more about why you think this is driven more by right-wingers? Do you have any analyses to back this up?

Answer: We did not want to suggest that the effect is only driven by the right. Instead, from a perspective of taking climate action, the gap on the right can be seen as an opportunity to increase support for climate action by elevating right-leaning politicians' beliefs to the level of their voters. Whereas, on the left, where politicians' beliefs are already higher than among citizens, it would not be helpful to align their beliefs with left-leaning citizens. In our analysis, we do state that beliefs among right-leaning politicians are significantly lower, but we also acknowledge that politicians on the left have very strong beliefs, and stronger beliefs than left-leaning citizens. For comparison, we report that this difference between left and right is smaller among citizens. We now additionally report marginal means and contrasts at ±1 SD of political orientation for politicians and citizens and show the difference between politicians and their electorate, both on the left and the right side (see also our answer to reviewer 1 comment 3). We further acknowledge that although there is a stark difference on the left wing, both politicians and citizens are on the higher end of the scale, whereas on the right side, citizens and politicians show lower acceptance of anthropogenic climate change (lines 308-391). In the discussion, we put more focus on lower climate beliefs among the right, and the gap between politicians and citizens, since aligning beliefs and subsequently policy support among right-wing politicians with their constituents holds potential to increase climate action. Whereas

politicians and citizens on the left already have strong beliefs and policy support. We hope that our explanation and the further analyses clarify that we do not want to state that the polarization effects are driven more by the right, but instead want to highlight the potential for reducing polarization by increasing climate beliefs on the political right.

The revised manuscript now states in the result section (lines 308-391):

> "However, the two-way interaction between political orientation and politician/citizen status indicates that political polarization of belief in anthropogenic climate change was stronger among politicians than citizens (b = -0.12, β = -0.18, p < .001), as illustrated in Fig. 1. Simple slope analyses confirmed a stronger association of political orientation with belief in climate change among politicians (b = -0.35 and β = -0.53, p < .001) than among citizens (b = -0.12 and β = -0.18, p < .001) and the slopes differed significantly between those groups (t(18,932) = -7.118, p < .001). Estimated marginal means and contrasts at ±1 standard deviation from the sample mean of political orientation (2.86 and 7.6 on a scale from '0' to '10'; see Supplementary Table S9) further illustrate the divergence in the political orientation slope between politicians and citizens. While right-leaning citizens reported only slightly lower belief than left-leaning citizens (Δ = 0.56, t(18,932) = 17.307, p < .001), politicians exhibited a much steeper ideological divide, with substantially lower belief among right-leaning politicians and very strong belief among left-leaning politicians (Δ = 1.32, t(18,932) = 17.949, p < .001). This asymmetry was driven by divergence at both ends of the spectrum. On the left, politicians expressed stronger climate beliefs than citizens (Δ = 0.752, t(18,932) = 10.157, p < .001), though both were on the higher end of the scale ($M_{\text{Politicians,left}}$ = 6.21; $M_{\text{Citizens,left}}$ = 5.46). On the right, the pattern reversed: Politicians expressed weaker belief in climate change than citizens (Δ = -0.362, t(18,932) = -2.460, p = .014), with both groups above but closer to the scale midpoint ($M_{\text{Politicians,right}}$ = 4.53; $M_{\text{Citizens,right}}$ = 4.89), suggesting lower acceptance of human-driven climate change. As Fig. 1 illustrates, the gaps between politicians and their electorate widened towards the ideological extremes."

2. You have done a very convincing job in establishing that political elites are much more polarized than are citizens. In the discussion, it would be wonderful if the researchers spent more time explaining why they think this is the case. According to many researchers, citizens merely adopt the political views of elites and do not form political attitudes and beliefs on their own (unless they are politically sophisticated; e.g., Van Boven & Sherman, 2021; Sniderman & Bullock, 2004). The data here show that this might not always be the case, regardless of education/political

sophistication. Why? Are citizens capable of forming political beliefs without elite input? Is climate change a special issue in which elites influence public opinion to a lesser degree than other political issues? Or does it trickle down in other ways (e.g., specific beliefs may differ, but hostility – or affective polarization – among politicians and citizens mirror one another)? It would be great if the authors could touch more on these issues and also incorporate more literature regarding how citizens form their political opinions (e.g., bottom up vs top down).

Answer: We thank the reviewer for this helpful suggestion. In response, we expanded the discussion section to further elaborate on why political elites may be more polarized than citizens and how this relates to theories of top-down versus bottom-up opinion formation. Specifically, we now discuss how politicians face incentive structures (e.g., party discipline, electoral incentives, and lobbying pressures) that may encourage more extreme public position-taking independent of their private beliefs. We also clarify that elite cues are an important driver of public opinion, as we already established in the introduction citing e.g. Van Boven & Sherman, 2021, However, we also acknowledge that citizens form attitudes through multiple channels beyond elite messaging (e.g., socialization, personal experiences, and information from non-elite sources), which can attenuate the transmission of elite polarization to the mass public. This helps reconcile our findings with existing theories suggesting that citizens often rely on elite cues while still allowing for weaker polarization among the public.

The revised manuscript now states in the discussion section (lines 640-682):

> "However, beyond misperception of public opinion [34,35] or stronger ideological influences on issue attitudes [36], the stronger polarization of climate beliefs among politicians could also be explained by strategic position-taking rather than actual epistemic beliefs about climate change. Politicians face incentive structures such as party constraints, electoral incentives or lobbying pressures [37, 38] that citizens do not have when stating opinions, potentially leading to more extreme expressions of climate opinions regardless of their personal assessment of the science. Such polarized elite positions can shape voters' attitudes about climate change [11, 39] and drive ideological and affective polarization among the public as well, particularly among politically interested citizens [40, 41]. Our findings that politicians are more polarized than citizens are consistent with models of top-down opinion formation [15] predicting that elite cues influence citizen opinion, not that citizens will mirror elite positions in full. Citizens form political attitudes through multiple channels beyond elite cues, including socialization, issue salience, personal experience, other sources of information and self interest, which attenuates the degree to which elite-level polarization is transmitted to the public [42-44]. Still, increasingly partisan elite positions may have driven

> public skepticism towards climate science as well [12]. Addressing the political incentives and ideological constraints that are potentially driving elite polarization requires structural solutions alongside epistemic ones, such as reducing financial and lobbying incentives for climate skepticism, or reforming electoral systems to reduce re-election pressures that reward partisan positioning. Informing right-leaning citizens that their representatives' climate positions diverge from their own could further encourage them to pressure politicians or vote accordingly, increasing congruence between politicians' positions and voters' preferences."

We believe this addition clarifies how our results relate to existing literature on elite cueing and citizen opinion formation, and addresses the reviewer's suggestion to situate the findings more explicitly within theories of political attitude formation.

Results

3. In the main text (possibly in the figure for each country), please add the n for each country. Additionally, were any countries more represented than others? If so, is there a possibility that the results of the primary analyses reported and plotted in Figure 1a are more influenced by one country compared to the others? How are the authors dealing with this issue statistically? One potential solution could be to use a multi-level model.

Answer: We thank the reviewer for raising this important methodological point. Indeed, sample sizes varied across countries, particularly within the politician group. We moved the supplementary table giving an overview of sample sizes per country to the results section in the main text (Table 2). In our analyses, we address the concerns about unequal sample sizes through the following aspects:

Country was included as an effect-coded factor in all regression models. Effect coding ensures that all regression coefficients are interpreted relative to the grand mean across all countries rather than relative to a single reference country, and weights each country equally at the intercept level. The political orientation × politician interaction plotted in Figure 1a is interpreted relative to the grand mean across countries, with each country equally weighted at the intercept level. However, we acknowledge that the estimation of these slopes may be more influenced by countries with larger politician samples, which is why we additionally report the country-specific interactions in Figure 1b to demonstrate consistency of the pattern across countries. Furthermore, we use HC1 heteroscedasticity-robust standard errors throughout, which corrects for the heteroscedasticity introduced by unequal group sizes and ensures that our inference is not distorted by this imbalance. We now further clarify in the methods section that this approach also addresses unequal sample sizes between countries and not just between politicians and citizens (lines 944-953).

We agree that a multilevel model would, in principle, be an elegant solution. However, models including a random slope for the political orientation × group interaction - which is central to addressing the reviewer's concerns - failed to converge across all nine country groups, likely due to the limited number of higher-level units and small sample sizes in some countries. It is also debatable if multilevel models would lead to accurate inferences with a small number of upper-level units (e.g., see Elff et al., 2020, doi:10.1017/S0007123419000097). A random intercepts-only multilevel model converged and yielded fixed effect estimates consistent with our regression results, but as random intercepts-only models do not resolve the unequal sample size concern for slope estimates any better than our current approach, we do not consider it a more appropriate alternative. We therefore retain our effect-coded regression with robust standard errors as the primary analysis, which we believe adequately addresses the concern raised.

4. The way in which unstandardized and standardized betas are reported is slightly confusing – why do they have different p-values? If the p-values do not match, it means the coding of one or more variables is slightly off in the standardized analysis. Once that is fixed, the authors can provide one p-value that corresponds to both the unstandardized and standardized coefficients, which will read much smoother.

Answer: We thank the reviewer for raising this point. The p-values between unstandardized and standardized regressions slightly differed because we used scale-mean centered political orientation in the unstandardized regressions and path models, which is slightly lower than the grand-mean of the sample. We now use grand-mean centered political orientation in the unstandardized models, which directly corresponds to the standardized measure of political orientation. With this approach, the p-values are identical between unstandardized and standardized regression models and path analyses.

We have changed all unstandardized estimates reported in the main text, figures, and supplementary material and report the same p-values for both standardized and unstandardized models. Furthermore, we updated the description of the analyses in the method section (lines 978-982). However, despite using the grand-mean centered political orientation in the models, we use the original scale for political orientation in Figure 1, since it is more informative and easier to interpret in a graph. In addition, we now report estimates and p-values in the path analyses separately for each policy outcome to avoid confusion from reporting multiple estimates in a single term (lines 507-516 for politicians and lines 520-580 for citizens).

5. When describing the mediation, starting with the b path is confusing (e.g., the link between climate belief and policy support). Why not start with the total effect or the a path?

Answer: We thank the reviewer for pointing out issues with the reporting structure of the path analyses. We have restructured the results section for the path analyses so that it follows the logic of reporting a-path, b-path, direct effects, indirect effects, and total effects in that order for both politicians.

The revised manuscript now states in the results section on politicians (lines 437-471):

> "In the path analyses on policy support among politicians (N = 709 for increasing airplane ticket prices; N = 713 for subsidizing electric vehicles) shown in Fig. 2, belief in climate change is weaker among politicians with a right-leaning political orientation (coefficients for both policies were b = -0.33 and β = -0.54, all $p < .001$). Belief in anthropogenic climate change, however, had a strong and positive effect on support for mitigation policies (b = 0.59, β = 0.48, $p < .001$ for increasing airplane ticket prices and b = 0.32, β = 0.29, $p < .001$ for subsidizing electric vehicles). Political orientation only had a small direct effect on policy support, with right-leaning politicians showing less support for increasing airplane ticket prices (b = -0.10, β = -0.13, p = .001) and for subsidizing electric vehicles (b = -0.07, β = -0.11 , p = .013). However, political orientation had an indirect effect on policy support through belief in climate change (b = -0.19, β = -0.26, $p < .001$ for higher airplane ticket prices and b = -0.11, β = -0.16, $p < .001$ for subsidizing electric vehicles), suggesting that politicians' right-leaning orientation indirectly reduces policy support via a weaker belief in climate change. Consequently, political orientation has a significant total effect on policy support among politicians (b = -0.29, β = -0.39, $p < .001$ for higher airplane ticket prices and b = -0.18, β = -0.26, $p < .001$ for subsidizing electric vehicles) with a partial mediation through belief in climate change. As already observed in our preceding analysis, belief in climate change is weaker among right-leaning politicians. This weaker belief, in turn, diminishes the positive influence that belief in climate change has on support for both policies."

The revised manuscript now states in the results section on citizens (lines 520-551):

> "In the path analyses on citizens' policy support (N = 17,696 for increasing airplane ticket prices and N = 17,635 for subsidizing electric vehicles), the effect of political orientation on belief in climate change was smaller compared to politicians (b = −0.12, β = -0.17, $p < .001$ for increasing airplane ticket prices and b = −0.11, β = -0.17, $p < .001$ for subsidizing electric vehicles). However, similarly to politicians, belief in anthropogenic climate change showed a meaningful effect on policy support (b = 0.27, β = 0.22, $p < .001$ for increasing airplane ticket prices and b = 0.29, β = 0.25, $p < .001$ for subsidizing electric vehicles). The direct effect of political orientation on policy support was small for support

of increasing airplane ticket prices (b = -0.07, β = -0.09, p = < .001) and very small for support of subsidizing electric vehicles (b = -0.02, β = -0.03, p = <.001). The indirect effect of political orientation on both policy support items through belief in climate change was also very small in size although statistically significant (b = −0.03, β = −0.03, p < .001 for increasing airplane ticket prices and b = −0.03, β = −0.04, p < .001 for subsidizing electric vehicles). Among citizens, political orientation has only a small total effect on policy support (b = -0.10, β = -0.12, p < .001 for increasing airplane ticket prices and b = -0.06, β = -0.07, p < .001 for subsidizing electric vehicles). Although belief in climate change significantly mediates this relationship, the indirect effects are very small in magnitude, as shown in Fig. 2."

6. Please refrain from comparing the size of coefficients unless you are doing a direct test (e.g., page 9, where the authors claim "belief in anthropogenic climate change showed the strongest direct effect"). Please also be careful about using the word "direct effect" when talking about any path that is not the c'.

Answer: We thank the reviewer for this helpful clarification. We agree that comparing coefficient magnitudes without formal statistical tests may be misleading. Accordingly, we have removed statements comparing the size of effects within the regression models and path analyses. In addition, we have revised our terminology to avoid using the term "direct effect" when referring to the b-paths from climate beliefs to policy support.

7. The authors should be careful about claiming that an effect with a p-value of <.001 is negligible (e.g., page 9). There is no need to downplay the (very) significant indirect effects among citizens, given that the multi-group SEM indicates that the indirect effects are stronger among politicians.

Answer: We thank the reviewer for this important clarification. Our intention was not to suggest that the indirect effects among citizens are not significant, but rather that they are small in magnitude relative to conventional benchmarks and in comparison to the substantially larger indirect effects observed among politicians. To avoid conflating statistical significance with effect size, we have revised the manuscript to remove wording that could be interpreted as downplaying significant effects. We now explicitly state that the indirect effects among citizens are statistically significant but small in magnitude, and we emphasize the comparative difference in mediation strength between citizens and politicians.

The revised manuscript now states in the results section (lines 537-553):

> “The indirect effect of political orientation on both policy support items through belief in climate change was also very small in size although statistically significant (*b* = −0.03, *β* = −0.03, *p* < .001 for increasing airplane ticket prices and *b* = −0.03, *β* = −0.04, *p* < .001 for subsidizing electric vehicles). Among citizens, political orientation has only a small total effect on policy support (*b* = -0.10, *β* = -0.12, *p* < .001 for increasing airplane ticket prices and *b* = -0.06, *β* = -0.07, *p* < .001 for subsidizing electric vehicles). Although belief in climate change significantly mediates this relationship, the indirect effects are very small in magnitude, as shown in Fig. 2. In fact, mediation effects were substantially stronger among politicians.”

Minor comments:
a. Since the citizen and politicians samples were initially discussed independently, I assumed that separate analyses would be reported. Please state more clearly at the beginning of the results section that the primary analyses are conducted on a sample that includes both citizens and politicians.

Answer: We thank the reviewer for raising this point about reporting our primary analyses, and we agree that not explicitly mentioning the sample for regression analyses right from the start might be misleading. We have clarified at the beginning of the sample description that the, although we use a conjoint sample in the analyses, we describe the politician and citizen samples separately first (lines 162-166):

> “Although the primary analyses of this study are conducted with a sample containing both citizens and politicians to compare effects between them, we describe the citizen and politician samples separately in this section.”

Furthermore, we now explicitly state at the beginning of reporting the regression analyses (lines 281-288) that we use a conjoint sample and want to compare effects between politicians and citizens in these regression analyses:

> “We conducted unstandardized and standardized regression analyses on a sample that includes both politicians and citizens to predict the impact of political orientation, education, and the interaction of the two on belief in anthropogenic climate change and compare those effects between politicians and citizens across countries (*N* = 18,995; see Supplementary Tables S6-S7 for detailed results).”

b. It would be great if the authors added more information about the sample in the main text (as opposed to the method), particularly the impressive politician sample. For example, how they were recruited, and what level of government they are in.

Answer: We thank the reviewer for pointing out this opportunity to describe the politician sample in more detail. We now include more information about the level of government and recruitment directly at the beginning of the politician sample description in the results section. Furthermore, we added Supplementary Tables S3 and S4 to give detailed information about the representativeness of cooperating politicians compared to their parliaments regarding gender, age, seniority and party ideology.

The revised manuscript now states in the results section (lines 195-215):

> "All country teams of this project (except for Germany) made efforts to reach out to all national members of parliament via e-mail. In Germany, given the large parliament size, a sampled population of members of parliament at the national level was targeted, ensuring representativeness of parliament in terms of gender, party, and incumbent status. In addition, Belgium, a federalized country, included regional politicians in their target populations from Flanders and Wallonia. Supplementary Tables S3 and S4 display the representativeness of the full politician sample compared to their respective countries' parliaments regarding gender, age, seniority, and party ideology. Gender and age distributions in our sample are similar to the target population with few exceptions (e.g. higher percentage of females in Israel). Seniority is overall slightly lower than in the respective parliaments, and we have a slightly lower participation rate among the right-wing in some countries."

In addition, we now also mention the representativeness of the citizen sample for the respective country's general population regarding gender, age and education, drawn from the official national statistics for each country (lines 170-174). We also mention more details about the study procedure for the politician sample in the methods section (lines 841-846).

c. Please state more clearly when you are reporting a two or three-way interaction. For example, at the top of page 8, the authors say, "Although the difference in polarization between politicians and citizens varied across countries." It would be much clearer if they also described this as a three-way interaction between ideology, politician vs. citizen status, and country.

Answer: We thank the reviewer for pointing out the imprecise reporting of interaction effects. We now explicitly state the type of interaction we are reporting at the

beginning of the respective paragraphs in the results section for regression analyses (lines 308-313, lines 392-399, lines 409-413).

d. On page 6, the authors report that there is a significant interaction between political polarization and politician vs. citizen status. In the following sentence ("While right-leaning citizens show slightly lower belief than left-leaning citizens, belief in climate change was significantly lower"), it would be helpful if they could additionally report the simple effects of ideology on belief in climate change for both citizens and politicians (e.g., what are the slopes of the blue and red lines in Figure 1a). Is the following sentence ("Accordingly, the paired comparison of the political orientation effect resulted in a significant difference between the groups ($t(18726) = -7.118$, $p < .001$), with $b = -0.35$ and $\beta = -0.53$ (both $p < .001$) for politicians and $b = -0.12$ as well as $\beta = -0.18$ (both $p < .001$) for citizens") describing the simple effects? If so, can the authors state more clearly that these are simple effects meant to decompose the significant interaction?

Answer: We thank the reviewer for pointing this out, and we agree that the reporting of the simple effects should be made clearer. We now explicitly state that we are reporting simple slope analyses for both politicians and citizens right after reporting the overall interaction effect. Then we further report the direct comparison of these slopes between groups.

The revised manuscript now states in the results section (lines 313-371):

> "Simple slope analyses confirmed a stronger association of political orientation with belief in climate change among politicians ($b = -0.35$ and $\beta = -0.53$, p < .001) than among citizens ($b = -0.12$ and $\beta = -0.18$, p < .001), and the slopes differed significantly between those groups ($t(18{,}932) = -7.118$, $p < .001$). Estimated marginal means and contrasts at ±1 standard deviation from the sample mean of political orientation (2.86 and 7.6 on a scale from '0' to '10'; see Supplementary Table S9) further illustrate the divergence in the political orientation slope between politicians and citizens."

e. In the mediation figures, the a and the b paths specify the β, while the total effect only reports the standardized coefficient in parentheses.

Answer: We thank the reviewer for pointing out inconsistencies in Figure 2. We have added the β coefficients to the direct and total effects to ensure consistent reporting of coefficients across all paths.

f. Could the authors present the order of supplementary tables in the same order they are referenced in the main text (e.g., Table S10 is currently mentioned before Table S9)?

Answer: We thank the reviewer for pointing out the disconnect between the order of supplementary tables and their references in the text. We have realigned both structures so that the numbering of references in the text is in ascending order and the supplementary tables follow that order as well.

g. It is unclear to me why political extremity was controlled for, and how it was calculated. Was political extremity calculated using the political ideology measure, and if so, is it appropriate to use it in a model that also includes political ideology? It seems to be that the model would then contain redundant variables. A more in depth explanation would help clear this up.

Answer: We thank the reviewer for raising concerns regarding the introduction of political extremity into the regression models. For the exploratory analyses, we derived political extremity from the self-reported political orientation by mean-centering and squaring the political orientation measure. However, we agree that adding political extremity would contain redundant variables that complicate the interpretation of this model. Given these statistical issues and the lack of any impact on our results by this variable, we decided to omit political extremity from the exploratory analyses. We deleted the reportings on political extremity in the results, the measurement, and the analyses of the methods section. Furthermore, we updated the results of the exploratory regression analyses, including additional covariates without political extremity in Supplementary Tables S13 and S14.

If the authors can address these comments, I believe that the manuscript should be accepted.

Reviewer #3 (Remarks to the Author):

Thank you for the opportunity to review this well-written and impressive manuscript. It investigates the impact of political orientation on belief in human-driven climate change and support for mitigation policies among citizens and politicians in eight developed democracies. It compares the level of polarization of belief in anthropogenic climate change and policy support among the public and politicians, and it evaluates the impact of higher education levels among both groups as a proxy for science literacy. It nicely reviews the existing related literature to motivate its central research questions. The manuscript makes a compelling point that few

studies have explored the level of polarization among politicians compared to their constituencies on this topic, nor the relative impact of political orientation in driving belief polarization across a large sample of countries and politicians. The large cross-national dataset employed for the analyses is impressive, and the consistent finding that polarization is higher among politicians than the public is novel and important. The findings also demonstrate the powerful impact of political ideology on belief human-caused climate change for both groups, which is the strongest predictor of support for mitigation policies. The impact of political orientation on these beliefs is not conditional on education. Additionally, the analyses suggest that belief in human-driven climate change mediates the relationship between political orientation and support for mitigation policies for citizens and politicians. The results and analyses are clearly presented in the text and figures. The discussion section clearly addresses the implications of the findings for generating greater consensus for policy action among skeptical audiences. However, are there other ways in addition to emphasizing the scientific consensus on the topic to increase belief in human caused climate change? Is it worth mentioning the challenges of building consensus on polarized and politicized issues that can generate motivated reasoning and activate partisan group norms when people are processing scientific messages, and/or ways to break through this persistent ideological/partisan polarization? Overall, this manuscript makes a significant contribution to the existing literature on political polarization on climate change, and I look forward to its publication.

Answer: We thank the reviewer for this positive review of our manuscript and the interesting thoughts on expanding the implications for climate communication. We agree that motivated reasoning and partisan identities need to be addressed when discussing climate change as a politically polarized topic. We have expanded our section on informational interventions that reduced polarization in past literature while also acknowledging that partisan motivated cognition is an additional barrier that needs to be overcome, beyond merely educating about climate change. We mention successful approaches on reducing partisan identity impacts among citizens from past literature but we also state that such interventions still need to be tested on politicians, who we found to be even more polarized in our study.

The revised manuscript now states in the discussion section (lines 721-741):

> “Instead, recent literature has identified promising informational interventions for depolarizing climate change perceptions among the public, such as emphasizing effective collective action [9], reducing psychological distance of climate change impacts [9, 46], educating about climate science [47, 48], or communicating the scientific consensus on climate change [49, 50]. Beyond merely informing citizens, it is also important to overcome ideological barriers such as motivated partisan cognition and group identity norms [51]. Reducing the impact of partisan identities through framing climate action as value-congruent seems to be

a promising approach to reduce polarization of climate opinions [52–54]. However, given the even stronger polarization we found in this study among politicians and their political incentives possibly influencing climate opinions, it needs to be tested whether such interventions can also improve climate beliefs and policy support among political elites across the ideological spectrum.”

REVIEWERS' COMMENTS:

Reviewer #1 (Remarks to the Author):

I thank the authors for their thoughtful revisions. The revised manuscript is significantly strengthened, particularly in its engagement with political economy and elite behavior perspectives, additional analyses examining divergence across ideological groups, and the clearer acknowledgment of the limitations surrounding causal interpretation in the mediation analyses. Overall, I believe the authors have addressed my major concerns satisfactorily.
There are a couple areas that could possibly use some further attention or clarification if the authors are interested, but I would not say a full revision is necessary.
1. The revised results section adds a nuanced interpretation of the asymmetries across the ideological spectrum but still operationalizes polarization as linear slope - so comments such as in line 409: "The gaps between politicians and their electorate widen towards the ideological extremes" is more of a geometric property of two linear relationships with different slopes, rather than evidence of distinct nonlinear patterns of polarization. Perhaps future work by the authors could look more closely at non-linear relationships.
2. I still view some aspects of the mediation interpretation as theoretically constrained by the design, particularly given the possibility that policy positioning may shape expressed climate beliefs among politicians rather than the reverse. However, the revised manuscript now explicitly acknowledges this limitation and frames the mediation analyses more cautiously.
Thank you for the opportunity to review the revised version of this manuscript.

Answer: We thank the reviewer for their careful reading and kind assessment of our revisions, and for their constructive suggestions. We are glad that our revisions have addressed their major concerns satisfactorily.

Reviewer #2 (Remarks to the Author):

The paper has been much improved. I only have a few small comments:

1. The title for the section “Stronger polarization of belief in anthropogenic climate change among politicians” would be clearer if it said “Stronger polarization of belief in anthropogenic climate change among politicians compared to citizens” to make the comparison more salient in the section title.

Answer: We thank the reviewer for this suggestion for the section title, which we added in the manuscript.

2. Sometimes, between the b and the standardized b, the authors put the word “and”, which is not needed. Just list the coefficients.

Answer: We thank the reviewer for pointing this out. We removed the “and” throughout the results section where appropriate.

3. On page 9, when the authors say “However, the direction of the interaction effect was consistent across countries (see Supplementary Tables S11 and S12 for standardized slopes and contrasts between groups for each country),” they should further clarify this by also stating something along the lines of “in that slopes among politicians were steeper than those among citizens in all countries”.

Answer: We thank the reviewer for suggesting clarifications in the results. We added the suggestion to this sentence to further clarify the nature of the interaction across countries.

I believe that this paper should be accepted, given that these small changes are made.

Reviewer #3 (Remarks to the Author):

The authors have clearly responded to the suggestions and requests from all reviewers. I was enthusiastic about the original version of this manuscript, and the thoughtful suggestions and responses to the other reviewers' comments have resulted in an even stronger piece of research. I recommend it be published.

Answer: We thank the reviewer for their constructive feedback and their kind words.
